# Influence of flexoelectricity on coupled electromechanical response of 2D MXene/graphene-based hybrid piezocomposites

**DOI:** 10.1038/s41598-024-74507-0

**Published:** 2024-10-08

**Authors:** Kishor Balasaheb Shingare, Rohan B. Ambade, Nilesh Rajaram Chodankar, Mandar Rokhade, Amal Al Ghaferi, Andreas Schiffer, Kin Liao

**Affiliations:** 1 Department of Aerospace Engineering, Khalifa University Khalifa University of Science & Technology, Abu Dhabi, 127788 UAE; 2 Department of Mechanical and Nuclear Engineering, Khalifa University Khalifa University of Science & Technology, Abu Dhabi, 127788 UAE; 3 Knorr-Bremse Technology Center India Private Limited, Pune, 411057 India; 4 Rabdan Academy, Abu Dhabi, 127788 UAE; 5 Advanced Research & Innovation Center, Aerospace Engineering, Khalifa University of Science & Technology, Abu Dhabi, 127788 UAE

**Keywords:** MXene, Graphene, Piezo- and flexo-electricity, Piezocomposite, Micromechanics, Analytical and approximate solution, Engineering, Computational methods, Applied mathematics, Graphene, Nanoscale materials

## Abstract

This study investigates the influence of flexoelectricity on the coupled electromechanical behavior of MXene/graphene-based hybrid piezocomposite (MGHPC) plates. We developed an analytical model based on Navier’s solution and Kirchhoff’s plate theory, as well as an approximate model based on the Ritz method for validation purposes. A three-phase micromechanical modeling is developed for determining the effective properties of MGHPC composed of 2D MXene and graphene nano-reinforcements embedded in an epoxy matrix. These micromechanical models were implemented to predict the static and dynamic electromechanical response of MGHPC plates subject to various edge support and loading conditions. Both the analytical and approximate solutions provided unequivocal evidence of the profound impact of the flexoelectric effect on the bending and modal analysis of MGHPC nanoplates. The flexoelectric effect enhanced the stiffness of the nanoplate, irrespective of the support condition. This implies that MGHPC plates can be tailored for precise resonance frequencies and static deflection within nanoelectromechanical systems. This can be achieved by manipulating parameters such as boundary conditions and geometric attributes, including plate thickness/aspect ratio and graphene/MXene nano-reinforcements volume fractions.

## Introduction

Owing to their exceptional chemical and physical properties, two-dimensional (2D) materials, such as graphene, have garnered significant interest, rendering them apt for miscellaneous applications in a plethora of key technologies. Graphene possesses remarkably greater thermal and electrical conductivity, exceptional mechanical strength, and flexibility. The discovery of graphene has undoubtedly revolutionized the research area of materials science, paving the way for exploring and engineering other 2D materials with unique properties^1^. Several 2D materials, including hexagonal boron nitride (h-BN)^2^, phosphorene^3^, transition metal dichalcogenides (TMDs)^4^, antimonene^5^, layered metal oxides, layered double hydroxides, and many others^6^, were studied in recent decades. These materials have extraordinary electronic, optical, and mechanical properties, rendering them fit for various applications. The field of 2D materials has rapidly expanded, with researchers exploring synthesis methods to understand fundamental properties and potential applications. Currently, the research focuses on engineering materials with tailored properties by stacking distinct 2D materials to generate van der Waals heterostructures, forming new materials with exclusive properties and potential applications in electronics, energy, and sensing.

Among them, a recently developed category of layered 2D transition metal nitrides and/or carbides, known as “MXenes,” has emerged as a rapidly advancing field^7^. MXenes are primarily produced via the selected acid etching of the “A” layers from MAX phases, which are ternary compounds with a general configuration of $\:{\text{M}}_{\text{n}+1}\text{A}{\text{X}}_{\text{n}}$, whereas M signifies an early transition metal, A denotes a group IIIA or IVA element, X signifies carbides (C) and/or nitrides (N), and *n* = 1, 2, 3. MXenes, due to elevated electrical conductivity, exceptional mechanical properties, and tailorable surface chemistry, are potential candidates for energy storage, catalysis, sensors, and various applications^7,8^. $\:{\text{T}\text{i}}_{3}{\text{C}}_{2}{\text{T}}_{x}\:$MXene is the most explored of the MXene family, and active research efforts are being made to understand its properties and discover new applications^9,10^. 2D materials like nanofillers, nano-reinforcements, and nano-additives in composites have the potential to revolutionize various technologies and industries. Thus, it is crucial to find the effective properties of specific composites to ensure a structure’s safety, robustness, and sustainability. This process involves considering various parameters tailored to different static and structural analyses, gaining insight into the material’s performance and behavior. Recently, polymer-based composites reinforced with different 2D nanomaterials have become an exciting research interest among researchers because of their tailorable properties. In this, different polymers, such as polyamide, polyvinyl alcohol (PVA), polydiallyldimethylammonium chloride (PDDA), polyester, polyurethane, epoxy, etc., were studied in the existing literature. However, epoxy resin is the most commonly used polymer because of its characteristics and ease of availability^11–13^. In the context of polymer-based composites, Monastyreckis et al.^13^ used the finite element (FE) and the representative volume element (RVE) approach to explore the tensile strength and damage mechanism of MXene-epoxy composites by considering parameters including mechanical properties, particle size, and interfacial layer strength. They showed that a 30% volume fraction of aligned MXene gives higher characteristics (8.4 times Young’s modulus and 1.91 times tensile strength) compared to neat epoxy. Shingare et al.^14^ comprehensively reviewed the computational FE models for studying the mechanical properties of graphene-based rubber composites, considering the assumption of small deformation. Moreover, graphene-MXene-based hybrid composites offer a combination of the unique properties of both graphene and MXenes, making them highly advantageous in a wide range of applications. These composites combine the strengths of both materials, offering enhanced electrical, mechanical, and chemical properties. The ability to tailor their properties through fabrication techniques such as layer-by-layer (LbL) assembly^15^, cost-effective techniques like spray coating, solvent casting^16^, and hydrothermal synthesis followed by freeze-drying^17^ makes them adaptable to a wide range of industrial and technological challenges. Incorporating MXenes into matrix materials enhances electrical and thermal conductivity, mechanical strength, chemical stability, and ion storage capabilities. These benefits make MXene-based hybrid composites highly adaptable for diverse applications^18,19^, including energy storage, electronics, structural materials, thermal management, sensors, and catalysis. These unique properties of MXenes allow matrix materials to be customized for advanced technological needs. The existing literature lacks studies on piezoelectric composites integrated with 2D MXene and graphene nano-reinforcements to enhance their electromechanical properties. Understanding the electromechanical coupling phenomena is imperative for producing advanced micro-/nano-electromechanical systems (M-/NEMS) devices, and their effective properties can be utilized to investigate their behavior. This study highlights the importance of using micromechanical modeling for determining the effective piezoelastic constants of nano-reinforcement-based piezocomposites using a three-phase micromechanical model.

Recently, there has been a noticeable upswing in interest regarding the development of M-/NEMS using piezoelectric smart materials to create beams, wires, plates, membranes, and shell-like structures. These structures serve as smart actuators, sensors, capacitors, and generators for structural control and health monitoring applications^20–25^. Flexoelectricity and piezoelectricity are two phenomena that are closely related. Piezoelectricity refers to generating an electric response due to applied mechanical stress. Flexoelectricity represents a significant phenomenon, particularly in nano- and microscales^26^. This phenomenon involves the generation of electric polarization ($\:{P}_{i}$) resulting from strain gradient $\:\left({{\upepsilon\:}}_{\text{j}\text{k},\text{l}}\right)$ within all-dielectric materials, regardless of a non-centrosymmetric or a centrosymmetric structure. Illustratively, this relationship is written as follows:$\:\:{P}_{i}\approx\:\:{\text{d}}_{\text{i}\text{j}\text{k}}{{\upepsilon\:}}_{\text{j}\text{k}}+{{\upmu\:}}_{\text{i}\text{j}\text{k}\text{l}}^{\text{d}\text{i}\text{r}}{{\upepsilon\:}}_{\text{j}\text{k},\text{l}}$; where$\:\:{{\upmu\:}}_{\text{i}\text{j}\text{k}\text{l}}^{\text{d}\text{i}\text{r}}\:$and$\:\:{\text{d}}_{\text{i}\text{j}\text{k}}$ denote the direct flexoelectric and piezoelectric constants, respectively^27^. Sharma et al.^28^ observed a significant enhancement in resultant coupling when both flexoelectric and piezoelectric effects were present within the electrically poled material. These phenomena are critical for sensors, actuators, and energy-harvesting devices. For instance, Yang^29^ investigated the behavior of electroelastic plates when subjected to large electric fields using variational equations of electroelasticity. They focused on extensional and flexural motion while considering minor strain and cubic electric fields. Fernandes and Pouget^30^ proposed an efficient 2D piezoelectric plate model based on linear piezoelectricity theory. The efficacy of their plate model was validated with FE simulations. Notably, Maranganti et al.^31^ conveyed a considerable increase in the coupling when flexoelectric and piezoelectric effects were present in an electrically poled material. This was attributed to the significance of nanoscale piezoelectric plates as fundamental 2D elements in nanodevices. Yan and Jiang^32^ explored the influence of surface parameters on the electroelastic characteristics of thin nanosized piezoelectric plates subjected to electromechanical loadings. They incorporated a model with surface piezoelectric effect and general Young–Laplace equations. Liu et al.^33^ investigated the natural vibration of piezoelectric plates subjected to electro-thermo-mechanical loadings. They explored the Kirchhoff plate theory with non-local elasticity, highlighting the impacts of various parameters such as the aspect ratio, axial force, non-local parameter, temperature change, side-to-thickness ratio, and electric voltage on the natural vibration behavior of the plates. Mohammadi et al.^34^ studied the electromechanical characteristics of a heterogeneous flexoelectric membrane for energy harvesting and stretchable electronics applications. Mondal et al.^35^ analyzed smart functionally graded (FG) graphene-reinforced composite (GRC) plates to study their electromechanical characteristics using a higher-order shear deformation theory (HSDT) that did not consider flexoelectricity. Moreover, Singh et al.^36^ developed 3D FE models to examine the coupled electromechanical behavior of piezoelectric FG GRC actuators in single- and multi-layered configurations. Naskar et al.^37^ examined surface effects in piezoelectric graphene-reinforced FG nanocomposites using the extended Kantorovich method. Malikan and coauthors^38–40^ extensively studied different structural elements such as thin beams, plates, and shells, considering various effects, including piezoelectricity, piezomagneticity, flexoelectricity, flexomagneticity, and viscoelasticity. Malikan and Eremeyev^38^ investigated the combined effect of viscoelasticity, piezoelectricity, and flexoelectricity on nanobeams to study their dynamic response. In this, they used a nonlocal strain gradient elasticity model and the Kelvin–Voigt viscoelastic model to derive the natural frequencies of the nanobeam. Their findings suggested that viscoelastic coupling can significantly influence the flexoelectric properties, which has implications for the design of next-generation sensors, actuators, and M-/NEMS. Malikan et al.^39^ developed a 3D elasticity model for smart piezocomposite structures wherein they incorporated the thickness coordinate into the governing equations to accurately model the behavior of these materials, as traditional FE models didn’t consider the micro- and nano-sized solids. This study focused on creating and analyzing materials that combine the properties of composites with smart functionalities, such as piezoelectricity, flexoelectricity, and flexomagneticity. Most recently, Malikan^40^ developed a mathematical model based on modified Sander’s theory to investigate the mechanics of piezo-flexo-magneto-elastic nanocomposite doubly curved shells, considering lower and higher levels of electromagnetic fields. He demonstrated that doubly-curved shells with spherical revolutions can serve as high-strength engineering structures compared to flat plates. In addition to this, Yurkov and Yudin^41^ formulated a continuum model for thin plates, accounting for converse flexoelectric effects. They also derived analytical solutions for circular plates with round electrodes, showing that deflections are inversely proportional to the square of the plate thickness. Furthermore, flexoelectronics has become an emerging field that explores the electronic properties of materials under mechanical strain, particularly in centrosymmetric and non-centrosymmetric semiconductors like (ZnO, GaN, TiO_2,_ and Nb-SrTiO_3_)^42^. Due to their symmetric crystal structure, this field leverages the flexoelectric effect, where a strain gradient induces polarization in typically non-piezoelectric materials. Wang et al.^42^ proposed a new way to induce a piezoelectric-like response in different semiconductors by distorting their crystal structure through applied pressure. This approach allows for the mechanical switching of electronics on a nanoscale, opening up possibilities for strain-modulated electronics. Sun et al.^43^ used the concept of flexoelectricity to tune the mechanical behavior of silicon-based Schottky diodes. In this, they reported that applying a strain gradient can adjust the Schottky barrier height, rectification ratio, and other electrical properties of the diode. This was achieved through techniques like conductive atomic force microscopy (C-AFM), where the current-voltage characteristics of the diode are measured under different tip forces. Again, Sun et al.^44^ studied the induced polarization in piezoelectric semiconductor (PS) composite bilayers, revealing a size-dependent behavior due to the flexoelectric coupling effect. This significantly impacts the piezotronics effect in nano-thickness PS bilayers, offering valuable insights for designing novel piezoelectric semiconductor devices. Shang et al.^45^ explored a novel method to manipulate polar structures in wrinkled thin films using flexoelectricity. They demonstrated that by varying the wrinkle morphologies in these films, one could induce a transition from a nonpolar state to various polar patterns, such as uniaxial polar stripes, biaxial meronlike or antimeronlike structures, and polar labyrinths. Tian et al.^46^ proposed a new approach of compositional heterogeneity to enhance the flexoelectric response of BaTiO₃ (Barium Titanate) ceramics. This approach can significantly improve the material’s properties by introducing variations in composition (BaTiO_3_ and Zirconium dioxide - ZrO_2_), which can lead to enhanced dielectric and flexoelectric responses. Wu et al.^47^ proposed a bilayer MoS_2_-based metal-semiconductor field-effect transistors (MESFETs), which was fabricated using bilayer MoS_2_ on a Si/SiO2 substrate, with Pt as the gate electrode and Ag/Au as the source/drain electrodes. Combining traditional gate voltage, tip force, and the flexoelectric effect significantly enhances the device’s performance. For instance, the maximum carrier mobility achieved under these conditions is 470 cm²/(V·s), notably higher than using the gate voltage alone. Despite extensive research, a significant gap remains in the theoretical analysis of flexoelectricity in 2D MXene/graphene-based hybrid piezocomposites. Unlocking the complete potential of these materials needs further exploration of their piezoelectric properties, particularly flexoelectricity.

**Fig. 1:**
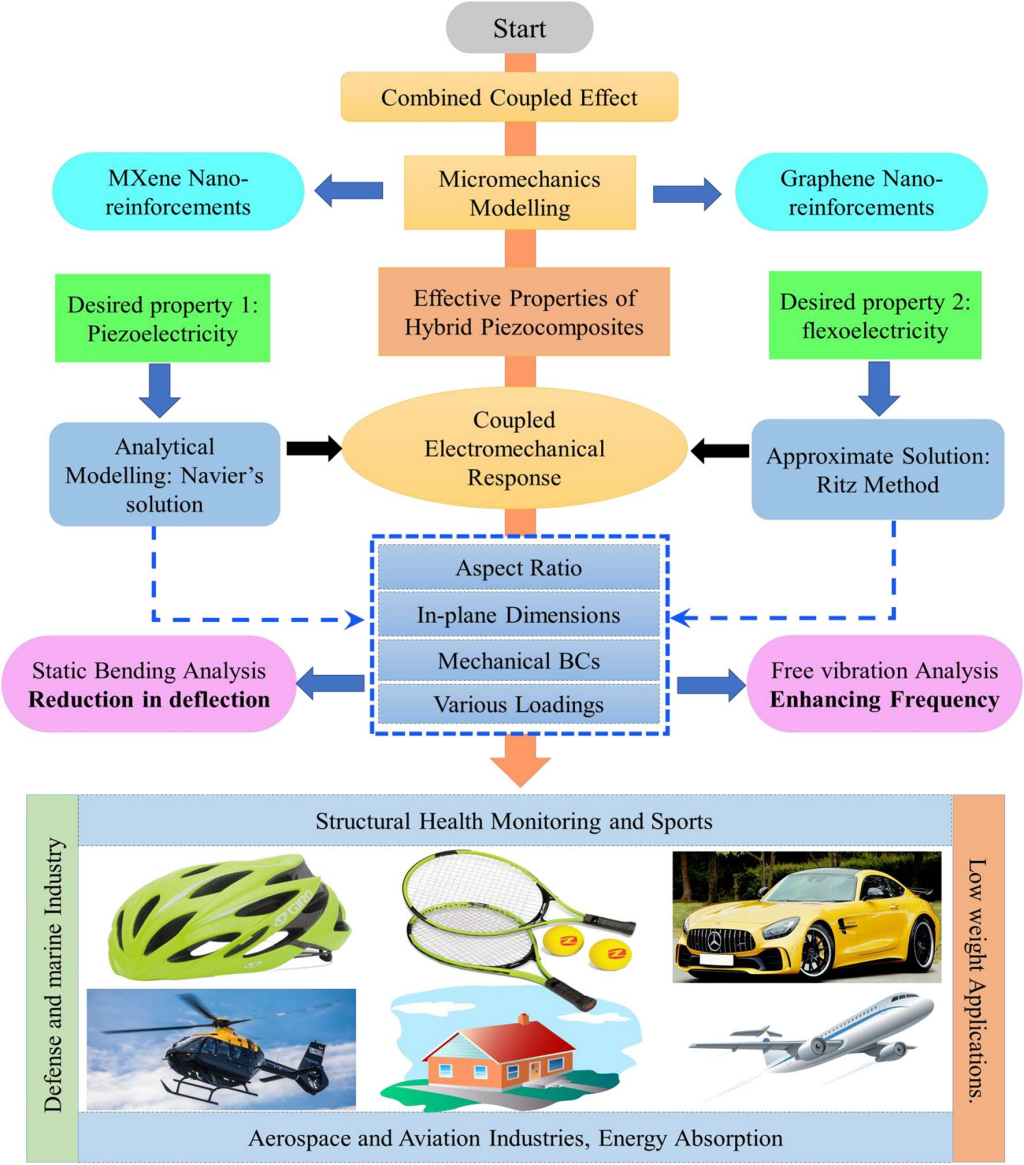
Flowchart illustrating the electromechanical behavior of MGHPC composite structures.

Recent research studies on graphene-based composite structures have primarily emphasized the utilization of graphene as nano-reinforcements. However, these research studies fail to account for these nano-reinforcements as piezoelectric materials that can be utilized in smart composite structures. There is a lack of studies on utilizing more than two nano-reinforcement composite structures, such as 2D MXene and graphene. The significant influence of flexoelectricity on the electromechanical characteristics of these novel structures has been neglected, which may lead to inaccurate outcomes. To address these shortcomings, we developed an exact analytical and approximate model that includes flexoelectricity and considered other nano-reinforcements such as graphene and MXene. To the best of the authors’ knowledge, no studies have examined the effect of flexoelectricity on 2D MXene/graphene-based hybrid piezocomposite (MGHPC) nanostructures subjected to different edge support conditions (e.g., simply supported (SSSS), clamped simply supported (CCSS), clamped-clamped (CCCC), and many more) and loadings (uniformly distributed load (UDL) and sinusoidal load). This study comprehensively considers influential factors for the analytical and approximate modeling of MGHPC nanoplate structures. This unexplored research area holds great potential for advancing next-generation M-/NEMS. Figure 1 illustrates the flowchart of the electromechanical behavior of MGHPC composite structures and its potential applications in various industries, including automotive, aerospace, aviation, and other engineering fields. Our study aims to contribute to understanding electric field and strain gradients in nanoplates, focusing on those exhibiting flexoelectric effects. We envisage that the present work advances our understanding of nanostructures and provides an opportunity to develop high-performance MXene-based nanoactuators and sensors.

## Electromechanical response of MXene/graphene-based piezocomposites

### Micromechanical model

**Fig. 2:**
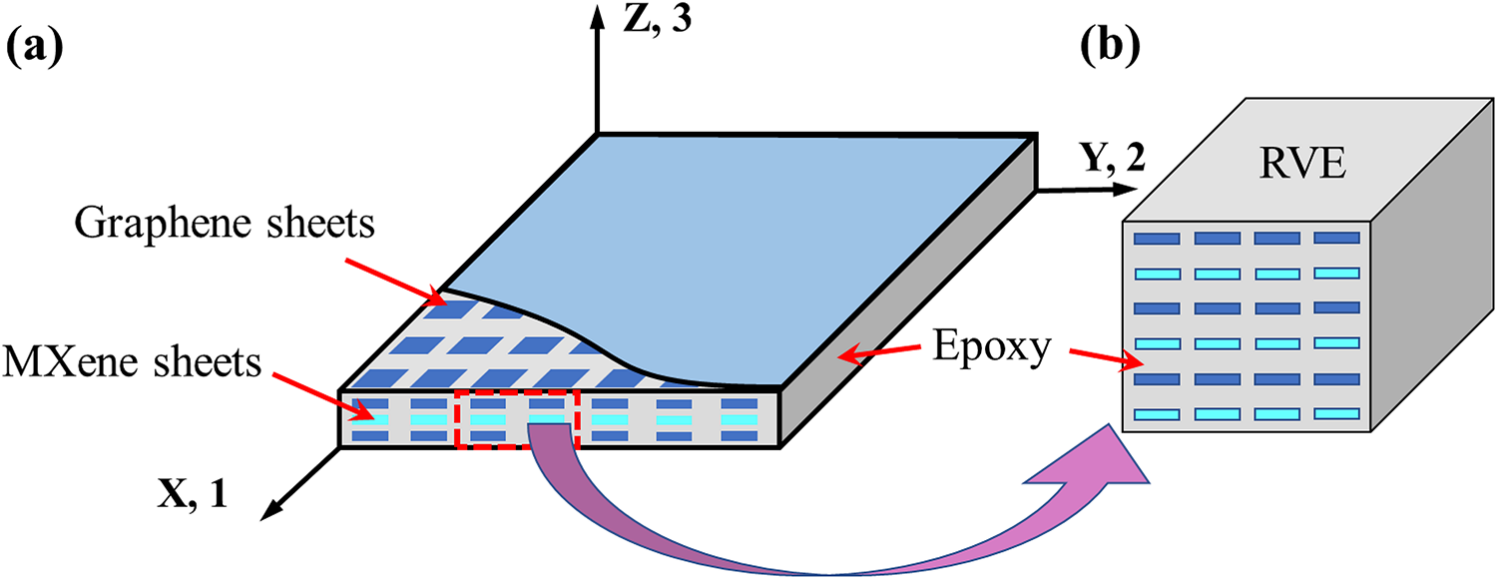
Schematic representation of three-phase RVE of *MGHPC* lamina.

The effective properties of piezoelectric MGHPC are determined by developing two- and three-phase micromechanical models considering graphene nano-reinforcements, MXene nano-reinforcements, and epoxy matrix. This distinction highlights the potential role of MXene and graphene as nanofillers or additives. In case of piezocomposites, Smith and Auld^48^ used the strength of materials model to predict the effective properties of a piezocomposite composed of PZT fibers and epoxy material. In this study, micromechanical models were formulated based on the concept of an RVE (refer to Fig. 2) to evaluate the composite lamina’s effective properties. Figure 2 depicts an RVE of piezoelectric graphene and MXene composite lamina embedded in the X- or 1- direction. The extracted RVE has properties equivalent to the lamina since the strain energy stored in both is almost identical.

The interaction between reinforcement and matrix components is crucial for optimizing composite material performance. This can be achieved through surface treatments applied to the reinforcement, matrix, or both. Sizing is widely used to enhance the compatibility between reinforcements and matrices. Nanofillers can enhance matrix adhesive properties, which captivated significant researchers’ attention^13,49–53^. This current research aims to strengthen composite materials’ performance by treating the epoxy matrix with MXene nanofillers and piezoelectric graphene nano-reinforcement. This strategic treatment is intended to optimize the interfacial characteristics between the matrix and the nanofillers.

The constitutive relation for the individual phase of the proposed three-phase piezocomposite can be expressed by Eq. (1).

1 $$\begin{aligned} & \left\{ {\upsigma ^{{{\text{pg}}}} } \right\} = \left[ {{\text{C}}^{{{\text{pg}}}} } \right]\left\{ {\upvarepsilon ^{{{\text{pg}}}} } \right\} - \left\{ {{\text{e}}^{{{\text{pg}}}} } \right\}{\text{E}}_{3} ; \\ & \left\{ {\upsigma ^{{\text{m}}} } \right\} = \left[ {{\text{C}}^{{\text{m}}} } \right]\left\{ {\upvarepsilon ^{{\text{m}}} } \right\}; \\ & \left\{ {\upsigma ^{{\text{e}}} } \right\} = \left[ {{\text{C}}^{{\text{e}}} } \right]\left\{ {\upvarepsilon ^{{\text{e}}} } \right\} \\ \end{aligned}$$

wherein

$$\begin{gathered} \left\{ {\upsigma ^{{\text{k}}} } \right\} = \left[ {\begin{array}{*{20}c} {\upsigma _{1}^{{\text{k}}} } & {\upsigma _{2}^{{\text{k}}} } & {\upsigma _{3}^{{\text{k}}} } & {\upsigma _{{23}}^{{\text{k}}} } & {\upsigma _{{13}}^{{\text{k}}} } & {\upsigma _{{12}}^{{\text{k}}} } \\ \end{array} } \right]^{\prime } ,\left\{ {\upvarepsilon ^{{\text{k}}} } \right\} = \left[ {\begin{array}{*{20}c} {\upvarepsilon _{1}^{{\text{k}}} } & {\upvarepsilon _{2}^{{\text{k}}} } & {\upvarepsilon _{3}^{{\text{k}}} } & {\upvarepsilon _{{23}}^{{\text{k}}} } & {\upvarepsilon _{{13}}^{{\text{k}}} } & {\upvarepsilon _{{12}}^{{\text{k}}} } \\ \end{array} } \right]^{\prime } \hfill \\ \left\{ {{\text{e}}^{{{\text{pg}}}} } \right\} = \left[ {\begin{array}{*{20}c} {{\text{e}}_{{31}}^{{{\text{pg}}}} } & {{\text{e}}_{{32}}^{{{\text{pg}}}} } & {{\text{e}}_{{33}}^{{{\text{pg}}}} } & 0 & 0 & 0 \\ \end{array} } \right]^{\prime } \hfill \\ \left[ {{\text{C}}^{{\text{k}}} } \right] = \left[ {\begin{array}{*{20}c} {{\text{C}}_{{11}}^{{\text{k}}} } & {{\text{C}}_{{12}}^{{\text{k}}} } & {{\text{C}}_{{13}}^{{\text{k}}} } & 0 & 0 & 0 \\ {{\text{C}}_{{12}}^{{\text{k}}} } & {{\text{C}}_{{22}}^{{\text{k}}} } & {{\text{C}}_{{23}}^{{\text{k}}} } & 0 & 0 & 0 \\ {{\text{C}}_{{13}}^{{\text{k}}} } & {{\text{C}}_{{23}}^{{\text{k}}} } & {{\text{C}}_{{33}}^{{\text{k}}} } & 0 & 0 & 0 \\ 0 & 0 & 0 & {{\text{C}}_{{44}}^{{\text{k}}} } & 0 & 0 \\ 0 & 0 & 0 & 0 & {{\text{C}}_{{55}}^{{\text{k}}} } & 0 \\ 0 & 0 & 0 & 0 & 0 & {{\text{C}}_{{66}}^{{\text{k}}} } \\ \end{array} } \right]{\text{k}} = pg,m,{\text{or}}\;e. \hfill \\ \end{gathered}$$

Here *pg*, *m*, and *e* signify the piezoelectric graphene sheets, MXene, and epoxy matrix. The superscript “k” specifies the different phases; $\:\left\{{{\upsigma\:}}^{\text{k}}\right\}$ and $\:\left\{{{\upepsilon\:}}^{\text{k}}\right\}$ signify the corresponding matrices for normal/shear stresses and strains; $\:\left[{\text{C}}^{\text{k}}\right]$ is the stiffness matrix of the k^th^ phase, and $\:\left\{{\text{e}}^{\text{p}\text{g}}\right\}$ denotes the piezoelectric matrix for graphene. $\:{\text{E}}_{3}\:$is electric field.

We assume that perfect bonding exists among graphene, MXene sheets, and the epoxy matrix and entails the following uniform stress and strain conditions^48^.

2 $$\:\left\{\begin{array}{c}{\varvec{\upepsilon\:}}_{11}^{\mathbf{p}\mathbf{g}}\\\:{{\upsigma\:}}_{22}^{\text{p}\text{g}}\\\:{{\upsigma\:}}_{33}^{\text{p}\text{g}}\\\:{{\upsigma\:}}_{23}^{\text{p}\text{g}}\\\:{{\upsigma\:}}_{13}^{\text{p}\text{g}}\\\:{{\upsigma\:}}_{12}^{\text{p}\text{g}}\end{array}\right\}=\left\{\begin{array}{c}{\varvec{\upepsilon\:}}_{11}^{\mathbf{m}}\\\:{{\upsigma\:}}_{22}^{\text{m}}\\\:{{\upsigma\:}}_{33}^{\text{m}}\\\:{{\upsigma\:}}_{23}^{\text{m}}\\\:{{\upsigma\:}}_{13}^{\text{m}}\\\:{{\upsigma\:}}_{12}^{\text{m}}\end{array}\right\}=\left\{\begin{array}{c}{\varvec{\upepsilon\:}}_{11}^{\mathbf{e}}\\\:{{\upsigma\:}}_{22}^{\text{e}}\\\:{{\upsigma\:}}_{33}^{\text{e}}\\\:{{\upsigma\:}}_{23}^{\text{e}}\\\:{{\upsigma\:}}_{13}^{\text{e}}\\\:{{\upsigma\:}}_{12}^{\text{e}}\end{array}\right\}=\left\{\begin{array}{c}{\varvec{\upepsilon\:}}_{11}^{\mathbf{H}\mathbf{C}}\\\:{{\upsigma\:}}_{22}^{\text{H}\text{C}}\\\:{{\upsigma\:}}_{33}^{\text{H}\text{C}}\\\:{{\upsigma\:}}_{23}^{\text{H}\text{C}}\\\:{{\upsigma\:}}_{13}^{\text{H}\text{C}}\\\:{{\upsigma\:}}_{12}^{\text{H}\text{C}}\end{array}\right\}$$

Utilizing the rule of mixture (ROM) condition, we derive the following equation:

3 $$\:{\text{v}}_{\text{p}\text{g}}\left\{\begin{array}{c}{\varvec{\upsigma\:}}_{11}^{\mathbf{p}\mathbf{g}}\\\:{{\upepsilon\:}}_{22}^{\text{p}\text{g}}\\\:{{\upepsilon\:}}_{33}^{\text{p}\text{g}}\\\:{{\upepsilon\:}}_{23}^{\text{p}\text{g}}\\\:{{\upepsilon\:}}_{13}^{\text{p}\text{g}}\\\:{{\upepsilon\:}}_{12}^{\text{p}\text{g}}\end{array}\right\}+{\text{v}}_{\text{m}}\left\{\begin{array}{c}{\varvec{\upsigma\:}}_{11}^{\mathbf{m}}\\\:{{\upepsilon\:}}_{22}^{\text{m}}\\\:{{\upepsilon\:}}_{33}^{\text{m}}\\\:{{\upepsilon\:}}_{23}^{\text{m}}\\\:{{\upepsilon\:}}_{13}^{\text{m}}\\\:{{\upepsilon\:}}_{12}^{\text{m}}\end{array}\right\}+{\text{v}}_{\text{e}}\left\{\begin{array}{c}{\varvec{\upsigma\:}}_{11}^{\mathbf{e}}\\\:{{\upepsilon\:}}_{22}^{\text{e}}\\\:{{\upepsilon\:}}_{33}^{\text{e}}\\\:{{\upepsilon\:}}_{23}^{\text{e}}\\\:{{\upepsilon\:}}_{13}^{\text{e}}\\\:{{\upepsilon\:}}_{12}^{\text{e}}\end{array}\right\}=\left\{\begin{array}{c}{\varvec{\upsigma\:}}_{11}^{\mathbf{H}\mathbf{C}}\\\:{{\upepsilon\:}}_{22}^{\text{H}\text{C}}\\\:{{\upepsilon\:}}_{33}^{\text{H}\text{C}}\\\:{{\upepsilon\:}}_{23}^{\text{H}\text{C}}\\\:{{\upepsilon\:}}_{13}^{\text{H}\text{C}}\\\:{{\upepsilon\:}}_{12}^{\text{H}\text{C}}\end{array}\right\}\:;\:\text{w}\text{h}\text{e}\text{r}\text{e}\:{\text{v}}_{\text{p}\text{g}}+{\text{v}}_{\text{m}}\text{\:+\:}{\text{v}}_{\text{e}}=1\:$$

Here the superscript “HC” specifies the quantities of hybrid piezocomposite, $\:{\text{v}}_{\text{p}\text{g}},{\text{v}}_{\text{m}}\text{\:and\:}{\text{v}}_{\text{e}}$ specify the volume fractions of graphene, MXene, and the epoxy matrix. Thus, using Eqs. (1–3), we can express the stress and strain vectors of the homogenized piezocomposite in terms of the corresponding vectors for the nano-reinforcements and matrix phases.

4 $$\begin{gathered} \underbrace {{\left\{ {\upsigma ^{{{\text{HC}}}} } \right\}}}_{\begin{subarray}{l} Stress\:in\:homogenized \\ hybrid\:piezocomposite \end{subarray} } = \underbrace {{\left[ {{\text{C}}_{1} } \right]\left\{ {\upvarepsilon ^{{{\text{pg}}}} } \right\}}}_{\begin{subarray}{l} Graphene \\ sheets \end{subarray} } + \underbrace {{\left[ {{\text{C}}_{2} } \right]\left\{ {\upvarepsilon ^{{\text{m}}} } \right\}}}_{\begin{subarray}{l} MXene \\ sheets \end{subarray} } + \underbrace {{\left[ {{\text{C}}_{3} } \right]\left\{ {\upvarepsilon ^{{\text{e}}} } \right\}}}_{\begin{subarray}{l} Epoxy \\ matrix \end{subarray} } - \underbrace {{\left\{ {{\text{e}}_{1} } \right\}{\text{E}}_{3} }}_{\begin{subarray}{l} Piezoelectric \\ effect \end{subarray} } \hfill \\ \left[ {{\text{C}}_{4} } \right]\left\{ {\upvarepsilon ^{{{\text{pg}}}} } \right\} - \left[ {{\text{C}}_{5} } \right]\left\{ {\upvarepsilon ^{{\text{m}}} } \right\} = \left\{ {{\text{e}}_{2} } \right\}{\text{E}}_{3} \hfill \\ \left\{ {\upvarepsilon ^{{{\text{HC}}}} } \right\} = \left[ {{\text{V}}_{1} } \right]\left\{ {\upvarepsilon ^{{{\text{pg}}}} } \right\} + \left[ {{\text{V}}_{2} } \right]\left\{ {\upvarepsilon ^{{\text{m}}} } \right\} + \left[ {{\text{V}}_{3} } \right]\left\{ {\upvarepsilon ^{{\text{e}}} } \right\}\:{\text{and}} \hfill \\ \left[ {{\text{C}}_{5} } \right]\left\{ {\upvarepsilon ^{{{\text{pg}}}} } \right\} - \left[ {{\text{C}}_{6} } \right]\left\{ {\upvarepsilon ^{{\text{e}}} } \right\} = 0 \hfill \\ \end{gathered}$$

wherein

$$\:\left[{\text{C}}_{1}\right]=\left[\begin{array}{cccccc}{\text{v}}_{\text{p}\text{g}}{\text{C}}_{11}^{\text{p}\text{g}}&\:{\text{v}}_{\text{p}\text{g}}{\text{C}}_{12}^{\text{p}\text{g}}&\:{\text{v}}_{\text{p}\text{g}}{\text{C}}_{13}^{\text{p}\text{g}}&\:0&\:0&\:0\\\:{\text{C}}_{12}^{\text{p}\text{g}}&\:{\text{C}}_{22}^{\text{p}\text{g}}&\:{\text{C}}_{23}^{\text{p}\text{g}}&\:0&\:0&\:0\\\:{\text{C}}_{13}^{\text{p}\text{g}}&\:{\text{C}}_{23}^{\text{p}\text{g}}&\:{\text{C}}_{33}^{\text{p}\text{g}}&\:0&\:0&\:0\\\:0&\:0&\:0&\:{\text{C}}_{44}^{\text{p}\text{g}}&\:0&\:0\\\:0&\:0&\:0&\:0&\:{\text{C}}_{55}^{\text{p}\text{g}}&\:0\\\:0&\:0&\:0&\:0&\:0&\:{\text{C}}_{66}^{\text{p}\text{g}}\end{array}\right],\left[{\text{C}}_{2}\right]={\text{v}}_{\text{m}}\left[\begin{array}{cccccc}{\text{C}}_{11}^{\text{m}}&\:{\text{C}}_{12}^{\text{m}}&\:{\text{C}}_{13}^{\text{m}}&\:0&\:0&\:0\\\:0&\:0&\:0&\:0&\:0&\:0\\\:0&\:0&\:0&\:0&\:0&\:0\\\:0&\:0&\:0&\:0&\:0&\:0\\\:0&\:0&\:0&\:0&\:0&\:0\\\:0&\:0&\:0&\:0&\:0&\:0\end{array}\right],$$

$$\:\left[{\text{C}}_{3}\right]={\text{v}}_{\text{e}}\left[\begin{array}{cccccc}{\text{C}}_{11}^{\text{e}}&\:{\text{C}}_{12}^{\text{e}}&\:{\text{C}}_{13}^{\text{e}}&\:0&\:0&\:0\\\:0&\:0&\:0&\:0&\:0&\:0\\\:0&\:0&\:0&\:0&\:0&\:0\\\:0&\:0&\:0&\:0&\:0&\:0\\\:0&\:0&\:0&\:0&\:0&\:0\\\:0&\:0&\:0&\:0&\:0&\:0\end{array}\right], \:\left[{\text{C}}_{4}\right]=\left[\begin{array}{cccccc}1&\:0&\:0&\:0&\:0&\:0\\\:{\text{C}}_{12}^{\text{p}\text{g}}&\:{\text{C}}_{22}^{\text{p}\text{g}}&\:{\text{C}}_{23}^{\text{p}\text{g}}&\:0&\:0&\:0\\\:{\text{C}}_{13}^{\text{p}\text{g}}&\:{\text{C}}_{23}^{\text{p}\text{g}}&\:{\text{C}}_{33}^{\text{p}\text{g}}&\:0&\:0&\:0\\\:0&\:0&\:0&\:{\text{C}}_{44}^{\text{p}\text{g}}&\:0&\:0\\\:0&\:0&\:0&\:0&\:{\text{C}}_{55}^{\text{p}\text{g}}&\:0\\\:0&\:0&\:0&\:0&\:0&\:{\text{C}}_{66}^{\text{p}\text{g}}\end{array}\right]$$

$$\:\left[{\text{C}}_{5}\right]=\left[\begin{array}{cccccc}1&\:0&\:0&\:0&\:0&\:0\\\:{\text{C}}_{12}^{\text{m}}&\:{\text{C}}_{22}^{\text{m}}&\:{\text{C}}_{23}^{\text{m}}&\:0&\:0&\:0\\\:{\text{C}}_{13}^{\text{m}}&\:{\text{C}}_{23}^{\text{m}}&\:{\text{C}}_{33}^{\text{m}}&\:0&\:0&\:0\\\:0&\:0&\:0&\:{\text{C}}_{44}^{\text{m}}&\:0&\:0\\\:0&\:0&\:0&\:0&\:{\text{C}}_{55}^{\text{m}}&\:0\\\:0&\:0&\:0&\:0&\:0&\:{\text{C}}_{66}^{\text{m}}\end{array}\right],\text{\:\:}\left[{\text{C}}_{6}\right]=\left[\begin{array}{cccccc}1&\:0&\:0&\:0&\:0&\:0\\\:{\text{C}}_{12}^{\text{e}}&\:{\text{C}}_{22}^{\text{e}}&\:{\text{C}}_{23}^{\text{e}}&\:0&\:0&\:0\\\:{\text{C}}_{13}^{\text{e}}&\:{\text{C}}_{23}^{\text{e}}&\:{\text{C}}_{33}^{\text{e}}&\:0&\:0&\:0\\\:0&\:0&\:0&\:{\text{C}}_{44}^{\text{e}}&\:0&\:0\\\:0&\:0&\:0&\:0&\:{\text{C}}_{55}^{\text{e}}&\:0\\\:0&\:0&\:0&\:0&\:0&\:{\text{C}}_{66}^{\text{e}}\end{array}\right]$$

$$\left\{ {{\text{e}}_{1} } \right\} = \left\{ {\begin{array}{*{20}c} {{\text{v}}_{{{\text{pg}}}} {\text{e}}_{{31}}^{{{\text{pg}}}} } & {{\text{e}}_{{32}}^{{{\text{pg}}}} } & {{\text{e}}_{{33}}^{{{\text{pg}}}} } & 0 & 0 & 0 \\ \end{array} } \right\}^{\prime } ,\left\{ {{\text{e}}_{2} } \right\} = \left\{ {\begin{array}{*{20}c} 0 & {{\text{e}}_{{32}}^{{{\text{pg}}}} } & {{\text{e}}_{{33}}^{{{\text{pg}}}} } & 0 & 0 & 0 \\ \end{array} } \right\}^{\prime }$$

$$\left[ {{\text{V}}_{1} } \right] = \left[ {\begin{array}{*{20}c} 1 & 0 & 0 & 0 & 0 & 0 \\ 0 & {{\text{v}}_{{{\text{pg}}}} } & 0 & 0 & 0 & 0 \\ 0 & 0 & {{\text{v}}_{{{\text{pg}}}} } & 0 & 0 & 0 \\ 0 & 0 & 0 & {{\text{v}}_{{{\text{pg}}}} } & 0 & 0 \\ 0 & 0 & 0 & 0 & {{\text{v}}_{{{\text{pg}}}} } & 0 \\ 0 & 0 & 0 & 0 & 0 & {{\text{v}}_{{{\text{pg}}}} } \\ \end{array} } \right],\:\left[ {{\text{V}}_{{\text{2}}} } \right]{\text{ = }}\left[ {\begin{array}{*{20}c} {\text{0}} & 0 & 0 & 0 & 0 & 0 \\ {\text{0}} & {{\text{v}}_{{\text{m}}} } & 0 & 0 & 0 & 0 \\ {\text{0}} & 0 & {{\text{v}}_{{\text{m}}} } & 0 & 0 & 0 \\ {\text{0}} & 0 & 0 & {{\text{v}}_{{\text{m}}} } & 0 & 0 \\ {\text{0}} & 0 & 0 & 0 & {{\text{v}}_{{\text{m}}} } & 0 \\ {\text{0}} & 0 & 0 & 0 & 0 & {{\text{v}}_{{\text{m}}} } \\ \end{array} } \right]\:{\text{and}}$$

$$\:\left[{\text{V}}_{3}\right]=\left[\begin{array}{cccccc}0&\:0&\:0&\:0&\:0&\:0\\\:0&\:{\text{v}}_{\text{e}}&\:0&\:0&\:0&\:0\\\:0&\:0&\:{\text{v}}_{\text{e}}&\:0&\:0&\:0\\\:0&\:0&\:0&\:{\text{v}}_{\text{e}}&\:0&\:0\\\:0&\:0&\:0&\:0&\:{\text{v}}_{\text{e}}&\:0\\\:0&\:0&\:0&\:0&\:0&\:{\text{v}}_{\text{e}}\end{array}\right]$$

After substituting Eq. (1) into Eq. (4), the resulting constitutive equation for the piezocomposite lamina is expressed as:

5 $$\:\left\{{{\upsigma\:}}^{\text{H}\text{C}}\right\}=\left[{\text{C}}^{\text{H}\text{C}}\right]\left\{{{\upepsilon\:}}^{\text{H}\text{C}}\right\}-\left\{{\text{e}}^{\text{H}\text{C}}\right\}{\text{E}}_{3}$$

wherein $\:\left[{\text{C}}^{\text{H}\text{C}}\right]$ and $\:\left\{{\text{e}}^{\text{H}\text{C}}\right\}$ represent the effective elastic and piezoelectric matrices of the hybrid piezocomposite, respectively, and are expressed as follows:

6 $$\begin{gathered} \left[ {{\text{C}}^{{{\text{HC}}}} } \right] = \left[ {{\text{C}}_{7} } \right]\left[ {{\text{V}}_{6} } \right]^{{ - 1}} + \left[ {{\text{C}}_{1} } \right]\left[ {{\text{V}}_{5} } \right]^{{ - 1}} \hfill \\ \left\{ {{\text{e}}^{{{\text{HC}}}} } \right\} = \left\{ {{\text{e}}_{1} } \right\} + \left( {\left\{ {{\text{e}}_{2} } \right\}\left[ {{\text{C}}_{7} } \right]\left[ {{\text{V}}_{6} } \right]^{{ - 1}} \left[ {{\text{V}}_{1} } \right]\left[ {{\text{C}}_{4} } \right]^{{ - 1}} + \left\{ {{\text{e}}_{2} } \right\}\left[ {{\text{C}}_{1} } \right]\left[ {{\text{V}}_{5} } \right]^{{ - 1}} \left[ {{\text{V}}_{4} } \right]\left[ {{\text{C}}_{6} } \right]^{{ - 1}} } \right) \hfill \\ \end{gathered}$$

wherein

$$\begin{gathered} \left[ {{\text{C}}_{7} } \right] = \left[ {{\text{C}}_{2} } \right]\left[ {{\text{C}}_{5} } \right]^{{ - 1}} \left[ {{\text{C}}_{6} } \right] + \left[ {{\text{C}}_{3} } \right],\:\:\left[ {{\text{V}}_{{\text{4}}} } \right]{\text{ = }}\left[ {{\text{V}}_{{\text{2}}} } \right]\left[ {{\text{C}}_{{\text{5}}} } \right]^{{{\text{ - 1}}}} \left[ {{\text{C}}_{{\text{6}}} } \right]{\text{ + }}\left[ {{\text{V}}_{{\text{3}}} } \right] \hfill \\ \left[ {{\text{V}}_{5} } \right] = \left[ {{\text{V}}_{4} } \right]\left[ {{\text{C}}_{6} } \right]^{{ - 1}} \left[ {{\text{C}}_{4} } \right] + \left[ {{\text{V}}_{1} } \right],\:\left[ {{\text{V}}_{6} } \right] = \left[ {{\text{V}}_{1} } \right]\left[ {{\text{C}}_{4} } \right]^{{ - 1}} \left[ {{\text{C}}_{6} } \right] + \left[ {{\text{V}}_{4} } \right],\:{\text{and}} \hfill \\ \left\{ {{\text{e}}^{{{\text{HC}}}} } \right\} = \left\{ {\begin{array}{*{20}c} {{\text{e}}_{{31}}^{{{\text{HC}}}} } & {{\text{e}}_{{32}}^{{{\text{HC}}}} } & {{\text{e}}_{{33}}^{{{\text{HC}}}} } & 0 & 0 & 0 \\ \end{array} } \right\}^{\prime } . \hfill \\ \end{gathered}$$

### Electromechanical response of MXene/graphene-based piezocomposite plates

#### Governing equations for MGHPC plates

**Fig. 3:**
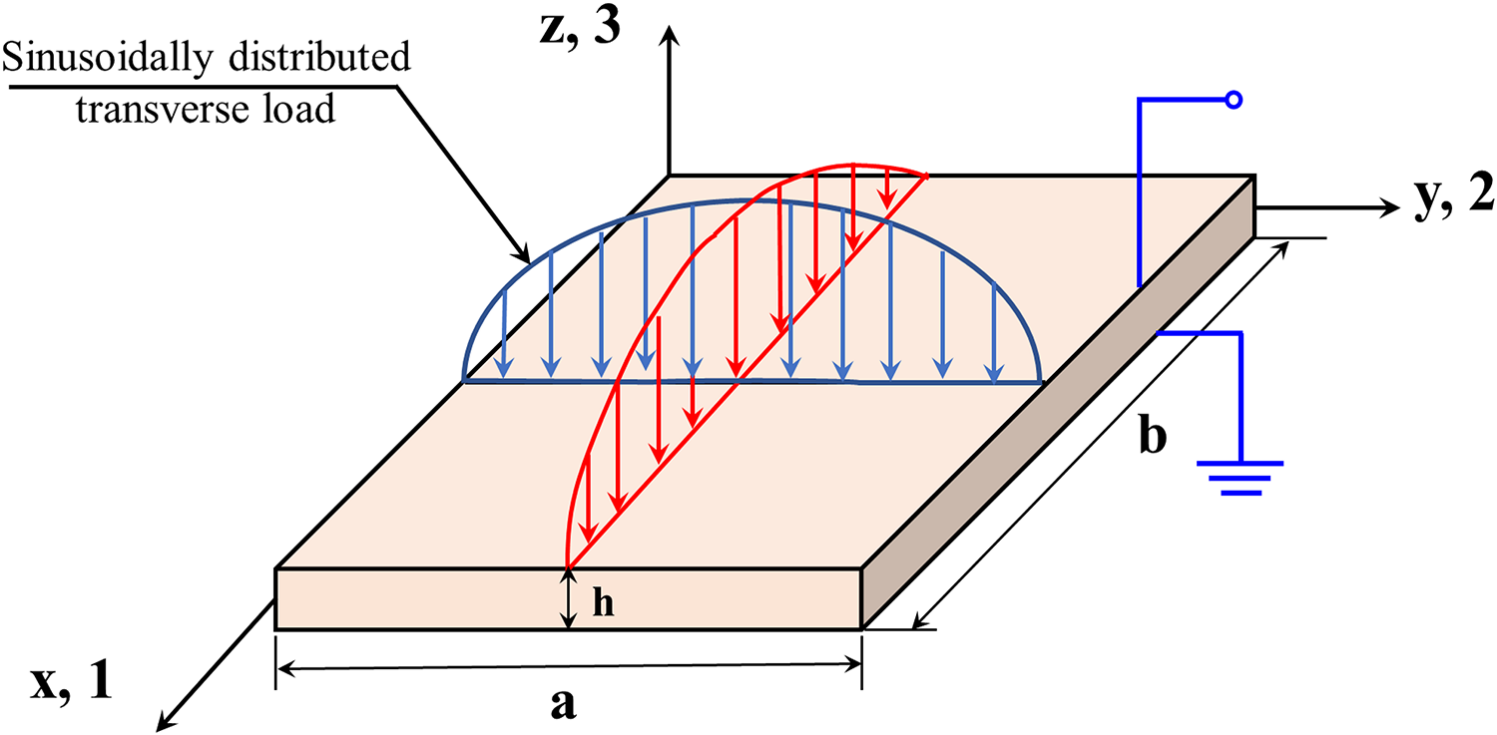
Representation of MGHPC plate under sinusoidal loading and open circuit condition.

Based on the micromechanical model, we developed an analytical and approximate model based on Navier solution and Ritz method, respectively, to envisage the static/dynamic response of a smart MGHPC plate (thickness *h*, width *b*, length *a*) subject to a sinusoidal load and under open circuit condition, as illustrated in Fig. 3. The Cartesian coordinate system defines the position of a material point within the plate, with thickness along the z-direction, while the undeformed nanoplate’s mid-plane aligns with the x-y plane.

According to Kirchhoff’s plate theory, the displacement $\:{\text{w}}_{0}(\text{x},\text{y},\text{t})$ of the plate in terms of transverse displacement can be expressed as^54^:

7 $$\:{\text{u}}_{0}\left(\text{x},\:\text{y},\:\text{z},\:\text{t}\right)=-\text{z}\frac{\partial\:{\text{w}}_{0}\left(\text{x},\:\text{y},\text{t}\right)}{\partial\:\text{x}};{\text{v}}_{0}\left(\text{x},\:\text{y},\:\text{z},\:\text{t}\right)=-\text{z}\frac{\partial\:{\text{w}}_{0}\left(\text{x},\:\text{y},\text{t}\right)}{\partial\:\text{y}};\:{\text{w}}_{0}\left(\text{x},\:\text{y},\:\text{z},\:\text{t}\right)={\text{w}}_{0}(\text{x},\text{y},\text{t})$$

where$\:\:{\text{u}}_{0}$ and $\:{\text{v}}_{0}$ denote the displacement in$\:\:\text{x}$ and$\:\:\text{y}$ directions, respectively; $\:\text{t}$ represents the time; and $\:{\text{w}}_{0}$ denotes the transverse displacement.

#### Assumptions of Kirchhoff’s plate theory^55^

- “Straight lines normal to the mid-surface (i.e., transverse normals) before deformation remains straight after deformation.”
- “The transverse normals are inextensible (i.e., do not experience elongation), and the plate thickness does not change during deformation.”
- “The transverse normals rotate such that they remain normal to the middle surface after deformation.”

The first two assumptions signify that transverse displacement ($\:{\text{w}}_{0}$) is not dependent on transverse (or thickness) coordinate, and transverse normal strain is zero ($\:{{\upepsilon\:}}_{\text{z}\text{z}}=0$). The last assumption indicates zero transverse shear strain ($\:{{\upepsilon\:}}_{\text{x}\text{z}}={{\upepsilon\:}}_{\text{y}\text{z}}=0$). Consequently, the nonzero strains are expressed as:

7a $$\:{{\upepsilon\:}}_{\text{x}\text{x}}=\:-\text{z}\frac{{\partial\:}^{2}{\text{w}}_{0}}{\partial\:{\text{x}}^{2}},\:\:{{\upepsilon\:}}_{\text{y}\text{y}}=\:-\text{z}\frac{{\partial\:}^{2}{\text{w}}_{0}}{\partial\:{\text{y}}^{2}},\:\:{{\upepsilon\:}}_{\text{x}\text{y}}=\:-\text{z}\frac{{\partial\:}^{2}{\text{w}}_{0}}{\partial\:\text{x}\partial\:\text{y}}$$

Assuming that the electric field $\:{\text{E}}_{\text{z}}$​ is solely present in the $\:\text{z}-$direction of the plate structure, it can be justified to eliminate the in-plane dimensions and electric field components in the $\:\text{x}-\text{y}$ plane by comparing them with those in the thickness direction^56^.

When assuming infinitesimal deformation and considering the electric field and strain gradient coupling, the electric Gibbs free energy density function (U) for centrosymmetric materials can be expressed by neglecting higher-order terms ($\:{\text{g}}_{\text{i}\text{j}\text{k}\text{l}\text{m}\text{n}}$ and $\:{\text{r}}_{\text{i}\text{j}\text{k}\text{l}\text{m}}$)^57^.

8 $${\text{U}} = - \frac{1}{2}\underbrace {{ \in _{{{\text{kl}}}} }}_{\begin{subarray}{l} Dielectric \\ term \end{subarray} }\underbrace {{{\text{E}}_{{\text{k}}} {\text{E}}_{{\text{l}}} }}_{\begin{subarray}{l} electric \\ field \end{subarray} } + \frac{1}{2}\underbrace {{{\text{C}}_{{{\text{ijkl}}}} }}_{\begin{subarray}{l} Elastic \\ term \end{subarray} }\underbrace {{\upvarepsilon _{{{\text{ij}}}} \upvarepsilon _{{{\text{kl}}}} }}_{{{\text{Strain}}}} - \underbrace {{{\text{e}}_{{{\text{ijk}}}} }}_{\begin{subarray}{l} Piezo \\ term \end{subarray} }\upvarepsilon _{{{\text{ij}}}} {\text{E}}_{{\text{k}}} - \underbrace {{{\text{f}}_{{{\text{ijkl}}}} }}_{\begin{subarray}{l} flexo \\ term \end{subarray} }{\text{E}}_{{\text{i}}} \underbrace {{\upeta _{{{\text{jkl}}}} }}_{\begin{subarray}{l} Strain \\ gradient \end{subarray} }$$

Thus, by taking flexoelectricity into account, the constitutive equations for a dielectric material can be expressed as^58,59^:

9 $$\:{{\upsigma\:}}_{\text{i}\text{j}}={\text{C}}_{\text{i}\text{j}\text{k}\text{l}}{{\upepsilon\:}}_{\text{k}\text{l}}-\:{\text{e}}_{\text{i}\text{j}\text{k}}{\text{E}}_{\text{k}}$$

10 $$\:{{\uptau\:}}_{\text{i}\text{j}\text{m}}=\:{-\text{f}}_{\text{k}\text{i}\text{j}\text{m}}{\text{E}}_{\text{k}}$$

11 $$\:{\text{D}}_{\text{i}}={\text{e}}_{\text{j}\text{k}\text{i}}{{\upepsilon\:}}_{\text{j}\text{k}}+\:{\in\:}_{\text{i}\text{j}}{\text{E}}_{\text{j}}+\:{\text{f}}_{\text{i}\text{j}\text{k}\text{l}}{{\upeta\:}}_{\text{j}\text{k}\text{l}}$$

$\:{{\uptau\:}}_{\text{i}\text{j}\text{m}}\:$denote higher-order stress tensor, in which the strain gradient tensor is also expressed as:

12 $$\:{{\upeta\:}}_{\text{j}\text{k}\text{l}}=\:{{\upepsilon\:}}_{\text{j}\text{k},\text{l}}$$

By employing the Eqs. (9–11), the constitutive relations are expressed as:

13a $$\:{{\upsigma\:}}_{\text{x}\text{x}}=\:{\text{C}}_{11}{{\upepsilon\:}}_{\text{x}\text{x}}+\:{\text{C}}_{12}{{\upepsilon\:}}_{\text{y}\text{y}}-\:{\text{e}}_{31}{\text{E}}_{\text{z}}$$

13b $$\:{{\upsigma\:}}_{\text{y}\text{y}}=\:{\text{C}}_{12}{{\upepsilon\:}}_{\text{x}\text{x}}+\:{\text{C}}_{11}{{\upepsilon\:}}_{\text{y}\text{y}}-\:{\text{e}}_{31}{\text{E}}_{\text{z}}$$

13c $$\:{{\uptau\:}}_{\text{x}\text{y}}=2{\text{C}}_{66}{{\upepsilon\:}}_{\text{x}\text{y}}$$

13d $$\:{{\uptau\:}}_{\text{x}\text{x}\text{z}}=\:{-\text{f}}_{14}{\text{E}}_{\text{z}}$$

13e $$\:{{\uptau\:}}_{\text{y}\text{y}\text{z}}=\:{-\text{f}}_{14}{\text{E}}_{\text{z}}$$

13f $$\:{\text{D}}_{\text{z}}=\:{\text{e}}_{31}\left({{\upepsilon\:}}_{\text{x}\text{x}}{+\:{\upepsilon\:}}_{\text{y}\text{y}}\right)+\:{\in\:}_{33}{\text{E}}_{\text{z}}+{\text{f}}_{14}\left({{\upeta\:}}_{\text{x}\text{x}\text{z}}{+\:{\upeta\:}}_{\text{y}\text{y}\text{z}}\right)$$

whereas $$\:{\text{f}}_{14}\:={\text{f}}_{1221}={\text{f}}_{2332}={\text{f}}_{1331}=\:{\text{f}}_{3113}$$$$\:=\:{\text{f}}_{3223}={\text{f}}_{2112}$$^60^.

From Eqs. (8, 10, 13d and 13e), it can be noticed that these higher-order stresses are induced by the flexoelectric effect, which is not present in the classical theory of elasticity and piezoelectricity^61^. In this formulation, the strain gradients, except for $\:{{\upeta\:}}_{\text{x}\text{x}\text{z}}$ and $\:{{\upeta\:}}_{\text{y}\text{y}\text{z}}$, are assumed to be zero for simplicity, as these specific strain gradients are considerably smaller compared to the strain gradients along the thickness direction of the MGHPC plate. With the absence of an external electric field, it becomes evident that the third term in Eq. (13f) signifies the polarization induced in the MGHPC plate because of the strain gradients.

Applying Gauss’ law of electrostatics and considering the absence of free electric charge, the electric displacement for the thin plate is expressed as:

14 $$\:\frac{\partial\:{\text{D}}_{\text{z}}}{\partial\:\text{z}}=0$$

Under open-circuit conditions (sensor type), the electric displacement over the surface of the plate is zero $\:\left({\text{D}}_{\text{z}}=0\right)$^62^, i.e., the electric voltage is zero. Consequently, the internal electric field $\:\left({\text{E}}_{\text{z}}\right)$ caused due to strain and strain gradient is obtained after putting Eq. (14) in Eq. (13f) as:

15 $${\text{E}}_{{\text{z}}} = \underbrace {{\frac{{{\text{e}}_{{31}} }}{{ \in _{{33}} }}}}_{\begin{subarray}{l} Piezoelectric \\ effect \end{subarray} }\left( {\frac{{\partial ^{2} {\text{w}}_{0} }}{{\partial {\text{x}}^{2} }} + \frac{{\partial ^{2} {\text{w}}_{0} }}{{\partial {\text{y}}^{2} }}} \right){\text{z}} + \underbrace {{\frac{{{\text{f}}_{{14}} }}{{ \in _{{33}} }}}}_{\begin{subarray}{l} flexoelectric \\ effect \end{subarray} }\left( {\frac{{\partial ^{2} {\text{w}}_{0} }}{{\partial {\text{x}}^{2} }} + \frac{{\partial ^{2} {\text{w}}_{0} }}{{\partial {\text{y}}^{2} }}} \right)$$

When the flexoelectric effect is considered, the internal electric field associated with piezoelectricity no longer maintains anti-symmetry to the midplane of the nanoplate in the direction of its thickness. It is observed that the $\:{\text{E}}_{\text{z}}$ is highly dependent on both the z-coordinate and flexoelectric coefficients. In the absence of piezoelectricity, the solution is solely attributed to the flexoelectric effect.

The equation that governs the plate problem is obtained using Hamilton’s variational principle^63^:

16 $$\:-{\updelta\:}{\int\:}_{\text{t}1}^{\text{t}2}\left({\int\:}_{\text{V}}^{\:}\text{U}\text{d}\text{V}+\text{K}+\text{W}\right)\text{d}\text{t}=0$$

wherein V denotes the total volume of the MGHPC plate. Under open-circuit conditions, the relation of U is expressed as^61^:

17 $$\:\text{U}=\:\frac{1}{2}{{\upsigma\:}}_{\text{i}\text{j}}{{\upepsilon\:}}_{\text{i}\text{j}}+\:\frac{1}{2}{{\uptau\:}}_{\text{i}\text{j}\text{k}}{{\upeta\:}}_{\text{i}\text{j}\text{k}}$$

Neglecting vibration along the $\:\text{x}-\text{y}$ plane, the kinetic energy (K) is expressed as:

18 $$\:\text{K}=\:\frac{1}{2}{\int\:}_{\text{V}}^{\:}{\uprho\:}{\left(\frac{\partial\:{\text{w}}_{0}}{\partial\:\text{t}}\right)}^{2}\text{d}\text{V}$$

whereas ρ denotes the density.

As a result of applying an external load, the work done (W) can be obtained as follows:

19 $$\:\text{W}=\:{\int\:}_{0}^{\text{a}}{\int\:}_{0}^{\text{b}}{\text{q}}_{0}\text{w}\text{d}\text{y}\text{d}\text{x}$$

Hence, the equation of motion governing bending deflection for any plate is expressed as:

20 $$\:\frac{{\partial\:}^{2}{\text{M}}_{\text{x}\text{x}}}{\partial\:{\text{x}}^{2}}+\frac{{\partial\:}^{2}{\text{M}}_{\text{x}\text{y}}}{\partial\:\text{x}\partial\:\text{y}}+\frac{{\partial\:}^{2}{\text{M}}_{\text{y}\text{x}}}{\partial\:\text{x}\partial\:\text{y}}+\frac{{\partial\:}^{2}{\text{M}}_{\text{y}\text{y}}}{\partial\:{\text{y}}^{2}}+\frac{{\partial\:}^{2}{\text{N}}_{\text{x}\text{x}\text{z}}}{\partial\:{\text{x}}^{2}}+\frac{{\partial\:}^{2}{\text{N}}_{\text{y}\text{y}\text{z}}}{\partial\:{\text{y}}^{2}}-{\uprho\:}\text{h}\frac{{\partial\:}^{2}{\text{w}}_{0}}{\partial\:{\text{t}}^{2}}+{q}_{0}=0$$

Therefore, one can determine the transverse deflection $\:\left({\text{w}}_{0}\right)$ of the plate by determining the bending moments $\:({\text{M}}_{\text{x}\text{x}},\:{\text{M}}_{\text{x}\text{y}},\:{\text{M}}_{\text{y}\text{x}}\:$and$\:\:{\text{M}}_{\text{y}\text{y}})$, and axial forces along the thickness $\:({\text{N}}_{\text{x}\text{x}\text{z}}\:$and$\:\:\:{\text{N}}_{\text{y}\text{y}\text{z}})$.

For example: In case of simply supported (SSSS) plates, boundary conditions prescribed on all four edges can be written as^55^:

21a $$\:\text{a}\text{t}\:\text{x}\:=0,\:\text{a}:\:{\text{w}}_{0}=0,\:{\text{M}}_{\text{x}\text{x}}=0.$$

21b $$\:\text{a}\text{t}\:\text{y}\:=0,\:\text{b}:\:{\text{w}}_{0}=0,\:{\text{M}}_{\text{y}\text{y}}=0.$$

The above bending moments and axial forces are determined as follows:

22a $$\:{\text{M}}_{\text{x}\text{x}}={\int\:}_{-\text{h}/2}^{\text{h}/2}{{\upsigma\:}}_{\text{x}\text{x}}\text{z}\text{d}\text{z},\:\:\:{\text{M}}_{\text{y}\text{y}}=\:{\int\:}_{-\text{h}/2}^{\text{h}/2}{{\upsigma\:}}_{\text{y}\text{y}}\text{z}\text{d}\text{z},\:{\text{M}}_{\text{x}\text{y}}={\text{M}}_{\text{y}\text{x}}=\:{\int\:}_{-\text{h}/2}^{\text{h}/2}{{\uptau\:}}_{\text{x}\text{y}}\text{z}\text{d}\text{z}$$

22b $$\:{\text{N}}_{\text{x}\text{x}\text{z}}=\:{\int\:}_{-\text{h}/2}^{\text{h}/2}{{\uptau\:}}_{\text{x}\text{x}\text{z}}\text{d}\text{z},\:\:{\text{N}}_{\text{y}\text{y}\text{z}}=\:{\int\:}_{-\text{h}/2}^{\text{h}/2}{{\uptau\:}}_{\text{y}\text{y}\text{z}}\text{d}\text{z}$$

By substituting Eqs. (7a) and (15) into the constitutive relations (13a–13e), the explicit expressions for the stresses and higher-order stresses associated with the transverse deflection $\:\left({\text{w}}_{0}\right)$ can be formulated as follows:

23a $$\:{{\upsigma\:}}_{\text{x}\text{x}}=\:-\left({\text{C}}_{11}+\:\frac{{\text{e}}_{31}^{2}}{{\in\:}_{33}}\right)\frac{{\partial\:}^{2}{\text{w}}_{0}}{\partial\:{\text{x}}^{2}}\text{z}-\left({\text{C}}_{12}+\:\frac{{\text{e}}_{31}^{2}}{{\in\:}_{33}}\right)\frac{{\partial\:}^{2}{\text{w}}_{0}}{\partial\:{\text{y}}^{2}}\text{z}-\frac{{\text{e}}_{31}{\text{f}}_{14}}{{\in\:}_{33}}\left(\frac{{\partial\:}^{2}{\text{w}}_{0}}{\partial\:{\text{x}}^{2}}+\:\frac{{\partial\:}^{2}{\text{w}}_{0}}{\partial\:{\text{y}}^{2}}\right)$$

23b $$\:{{\upsigma\:}}_{\text{y}\text{y}}=\:-\left({\text{C}}_{12}+\:\frac{{\text{e}}_{31}^{2}}{{\in\:}_{33}}\right)\frac{{\partial\:}^{2}{\text{w}}_{0}}{\partial\:{\text{x}}^{2}}\text{z}-\left({\text{C}}_{11}+\:\frac{{\text{e}}_{31}^{2}}{{\in\:}_{33}}\right)\frac{{\partial\:}^{2}{\text{w}}_{0}}{\partial\:{\text{y}}^{2}}\text{z}-\frac{{\text{e}}_{31}{\text{f}}_{14}}{{\in\:}_{33}}\left(\frac{{\partial\:}^{2}{\text{w}}_{0}}{\partial\:{\text{x}}^{2}}+\:\frac{{\partial\:}^{2}{\text{w}}_{0}}{\partial\:{\text{y}}^{2}}\right)$$

23c $$\:{{\uptau\:}}_{\text{x}\text{y}}=-2{\text{C}}_{66}\frac{{\partial\:}^{2}{\text{w}}_{0}}{\partial\:\text{x}\partial\:\text{y}}\text{z}$$

23d $$\:{{\uptau\:}}_{\text{x}\text{x}\text{z}}={{\uptau\:}}_{\text{y}\text{y}\text{z}}=-\frac{{\text{e}}_{31}{\text{f}}_{14}}{{\in\:}_{33}}\left(\frac{{\partial\:}^{2}{\text{w}}_{0}}{\partial\:{\text{x}}^{2}}+\frac{{\partial\:}^{2}{\text{w}}_{0}}{\partial\:{\text{y}}^{2}}\right)\text{z}-\frac{{\text{f}}_{14}^{2}}{{\in\:}_{33}}\left(\frac{{\partial\:}^{2}{\text{w}}_{0}}{\partial\:{\text{x}}^{2}}+\frac{{\partial\:}^{2}{\text{w}}_{0}}{\partial\:{\text{y}}^{2}}\right)$$

Utilizing Eqs. (23a–23d) in Eqs. (22a, 22b), the bending moments are obtained as follows:

24a $$\:{\text{M}}_{\text{x}\text{x}}=-\left[\left({\text{C}}_{11}+\frac{{\text{e}}_{31}^{2}}{{\in\:}_{33}}\right)\frac{{\partial\:}^{2}{\text{w}}_{0}}{\partial\:{\text{x}}^{2}}-\left({\text{C}}_{12}+\frac{{\text{e}}_{31}^{2}}{{\in\:}_{33}}\right)\frac{{\partial\:}^{2}{\text{w}}_{0}}{\partial\:{\text{y}}^{2}}\right]\frac{{\text{h}}^{3}}{12}$$

24b $$\:{\text{M}}_{\text{y}\text{y}}=-\left[\left({\text{C}}_{12}+\frac{{\text{e}}_{31}^{2}}{{\in\:}_{33}}\right)\frac{{\partial\:}^{2}{\text{w}}_{0}}{\partial\:{\text{x}}^{2}}-\left({\text{C}}_{11}+\frac{{\text{e}}_{31}^{2}}{{\in\:}_{33}}\right)\frac{{\partial\:}^{2}{\text{w}}_{0}}{\partial\:{\text{y}}^{2}}\right]\frac{{\text{h}}^{3}}{12}$$

24c $$\:{\text{M}}_{\text{x}\text{y}}={\text{M}}_{\text{y}\text{x}}=-\:\frac{2{\text{C}}_{66}{\text{h}}^{3}}{12}\:\frac{{\partial\:}^{2}{\text{w}}_{0}}{\partial\:\text{x}\partial\:\text{y}}$$

24d $$\:{\text{N}}_{\text{x}\text{x}\text{z}}={\text{N}}_{\text{y}\text{y}\text{z}}=-\frac{\text{h}{\text{f}}_{14}^{2}}{{\in\:}_{33}}\left(\frac{{\partial\:}^{2}{\text{w}}_{0}}{\partial\:{\text{x}}^{2}}+\:\frac{{\partial\:}^{2}{\text{w}}_{0}}{\partial\:{\text{y}}^{2}}\right)$$

Equations (23–24) highlight the importance of flexoelectricity in shaping stress and higher-order stress distribution. Consequently, the higher-order stress diminishes when the flexoelectric effect is not considered, leaving the conventional bending moments unaffected by the strain gradient polarization. Furthermore, when flexoelectricity is considered, the higher-order stresses are added together.

Equation (24) can be expressed in terms of $\:{\text{w}}_{0}$ by substituting Eq. (20) as:

25 $$\:{\text{G}}_{11}\left(\frac{{\partial\:}^{4}{\text{w}}_{0}}{\partial\:{\text{x}}^{4}}+\frac{{\partial\:}^{4}{\text{w}}_{0}}{\partial\:{\text{y}}^{4}}\right)+2\left({\text{G}}_{12}+2{\text{G}}_{66}\right)\frac{{\partial\:}^{4}{\text{w}}_{0}}{\partial\:{\text{x}}^{2}\partial\:{\text{y}}^{2}}+{\uprho\:}\text{h}\frac{{\partial\:}^{2}{\text{w}}_{0}}{\partial\:{\text{t}}^{2}}={q}_{0}$$

whereas

26 $$\:\left\{\begin{array}{c}{\text{G}}_{11}=\left({\text{C}}_{11}+\frac{{\text{e}}_{31}^{2}}{{\in\:}_{33}}\right)\frac{{\text{h}}^{3}}{12}+\frac{\text{h}{\text{f}}_{14}^{2}}{{\in\:}_{33}}\\\:{\text{G}}_{12}=\left({\text{C}}_{12}+\frac{{\text{e}}_{31}^{2}}{{\in\:}_{33}}\right)\frac{{\text{h}}^{3}}{12}+\frac{\text{h}{\text{f}}_{14}^{2}}{{\in\:}_{33}}\\\:{\text{G}}_{66}=\frac{{{\text{C}}_{66}\text{h}}^{3}}{12}\:\:\:\:\:\:\:\:\:\:\:\:\:\:\:\:\:\:\:\:\:\:\:\:\:\:\:\:\:\:\:\:\end{array}\right.$$

##### Solution for static response of MGHPC plates considering flexoelectricity

In case of static bending response of MGHPC plates, Eq. (25) can be re-formulated as follows^55,64^:

27 $$\:{\text{G}}_{11}\left(\frac{{\partial\:}^{4}{\text{w}}_{0}}{\partial\:{\text{x}}^{4}}+\frac{{\partial\:}^{4}{\text{w}}_{0}}{\partial\:{\text{y}}^{4}}\right)+\left({2\text{G}}_{12}+4{\text{G}}_{66}\right)\frac{{\partial\:}^{4}{\text{w}}_{0}}{\partial\:{\text{x}}^{2}\partial\:{\text{y}}^{2}}={q}_{0}$$

In the absence of flexoelectricity, Eq. (27) aligns with the conventional classical Kirchhoff plate theory, assuming linear piezoelectricity. According to conventional plate theory, Fourier series theory can be employed to determine $\:{\text{w}}_{0}(\text{x},\:\text{y})$ and solve the governing Eq. (27) of the SSSS MGHPC plate:

28 $$\:{\text{w}}_{0}\left(\text{x},\text{y}\right)=\sum\:_{\text{m}=1}^{{\infty\:}}\sum\:_{\text{n}=1}^{{\infty\:}}{\text{A}}_{\text{m}\text{n}}\text{s}\text{i}\text{n}{\upalpha\:}x\text{s}\text{i}\text{n}{\upbeta\:}y$$

whereas$\:\:{\upalpha\:}=\frac{\text{m}{\uppi\:}}{\text{a}},\:{\upbeta\:}=\frac{\text{n}{\uppi\:}}{\text{b}}$. The coefficients $\:{\text{A}}_{\text{m}\text{n}}$ are to be calculated for each m and n half wave number, ensuring satisfaction throughout the domain of a plate. It has been previously established that Eq. (28) satisfies the boundary conditions provided in Eq. (21). To calculate the uniformly distributed load $\:q\left(\text{x},\:\text{y}\right)={\text{q}}_{0}$ the Fourier series can be utilized as shown in Eq. (29a).

29a $$\:q\left(\text{x},\text{y}\right)=\sum\:_{\text{m}=1}^{{\infty\:}}\sum\:_{\text{n}=1}^{{\infty\:}}{\text{Q}}_{\text{m}\text{n}}\text{s}\text{i}\text{n}\alpha\:x\text{s}\text{i}\text{n}{\upbeta\:}\text{y}\:\:\:\:\:\:\:\:\:\:\:\:\:\:\:\:\:\:\:\:\:\:\:\:\text{a}\text{n}\text{d}$$

29b $$\:{\text{A}}_{\text{m}\text{n}}=\frac{{\text{Q}}_{\text{m}\text{n}}}{{\text{d}}_{\text{m}\text{n}}}$$

29c $$\:{\text{d}}_{\text{m}\text{n}}={\left(\frac{{\uppi\:}}{\text{b}}\right)}^{4}\left\{{\text{G}}_{11}{\left(\frac{\text{m}}{\text{a}}\right)}^{4}+{\text{G}}_{12}{\left(\frac{\text{n}}{\text{b}}\right)}^{4}+2\left({\text{G}}_{12}+2{\text{G}}_{66}\right){\left(\frac{\text{m}\text{n}}{\text{a}\text{b}}\right)}^{2}\right\}$$

Thus, the transverse deflection, $\:{\text{w}}_{0}\left(\text{x},\text{y}\right)$ can be obtained as:

30a $$\:{\text{w}}_{0}\left(\text{x},\text{y}\right)=\sum\:_{\text{m}=1}^{{\infty\:}}\sum\:_{\text{n}=1}^{{\infty\:}}\frac{{\text{Q}}_{\text{m}\text{n}}}{{\text{d}}_{\text{m}\text{n}}}\text{s}\text{i}\text{n}{\upalpha\:}x\text{s}\text{i}\text{n}{\upbeta\:}y$$

For a sinusoidally distributed load:

30b $$\:\text{q}\left(\text{x},\text{y}\right)={\text{q}}_{0}\text{s}\text{i}\text{n}\frac{{\uppi\:}\text{x}}{\text{a}}\:\text{s}\text{i}\text{n}\frac{{\uppi\:}\text{y}}{\text{a}}\:\:\:\:\:\:\:\:\:\:{\text{Q}}_{\text{m}\text{n}}={\text{q}}_{0}\:\text{a}\text{n}\text{d}\:\:\text{m}=\text{n}=1$$

While for UDL, $\:q\left(\text{x},\text{y}\right)={\text{q}}_{0}$ and hence, we have

30c $$\:{\text{Q}}_{\text{m}\text{n}}=\frac{16{\text{q}}_{0}}{\text{m}\text{n}{{\uppi\:}}^{2}}\:\:\:\:\:\:\:\:\:\:\:\:\:\:\:\:\:\:\:\:\:\text{a}\text{n}\text{d}\:\text{m},\:\text{n}=1,\:3,\:5\dots\:.$$

By making use of Eqs. (27–29), we can get exact solutions for transverse deflection, $\:{\text{w}}_{0}\left(\text{x},\text{y}\right)$ of simply supported MGHPC plates under UDL.

30d $$\:{\text{w}}_{0}\left(\text{x},\text{y}\right)=\sum\:_{\text{m}=1,\:\text{3,5}\dots\:}^{{\infty\:}}\sum\:_{\text{n}=1,\:\text{3,5}\dots\:}^{{\infty\:}}\frac{{\text{Q}}_{\text{m}\text{n}}\text{s}\text{i}\text{n}{\upalpha\:}x\text{s}\text{i}\text{n}{\upbeta\:}y}{{\left(\frac{{\uppi\:}}{\text{b}}\right)}^{4}\left\{{\text{G}}_{11}{\left(\frac{\text{m}}{\text{a}}\right)}^{4}+{\text{G}}_{12}{\left(\frac{\text{n}}{\text{b}}\right)}^{4}+2\left({\text{G}}_{12}+2{\text{G}}_{66}\right){\left(\frac{\text{m}\text{n}}{\text{a}\text{b}}\right)}^{2}\right\}}$$

##### Solution for dynamic response of MGHPC plates considering flexoelectricity

Using Eq. (25), we can determine the vibration response of the MGHPC plate as follows:

31 $$\:{\text{G}}_{11}\left(\frac{{\partial\:}^{4}{\text{w}}_{0}}{\partial\:{\text{x}}^{4}}+\frac{{\partial\:}^{4}{\text{w}}_{0}}{\partial\:{\text{y}}^{4}}\right)+2\left({\text{G}}_{12}+2{\text{G}}_{66}\right)\frac{{\partial\:}^{4}{\text{w}}_{0}}{\partial\:{\text{x}}^{2}\partial\:{\text{y}}^{2}}+\:{\uprho\:}\text{h}\frac{{\partial\:}^{2}{\text{w}}_{0}}{\partial\:{\text{t}}^{2}}=0$$

Analogous to classical plate model, the harmonic solution for $\:{\text{w}}_{0}\:(\text{x},\:\text{y},\text{t})$ can be expressed as:

32 $$\:{\text{w}}_{0}\left(\text{x},\text{y},\text{t}\right)=\sum\:_{\text{m}=1}^{{\infty\:}}\sum\:_{\text{n}=1}^{{\infty\:}}{\text{B}}_{\text{m}\text{n}}\text{s}\text{i}\text{n}\frac{\text{m}{\uppi\:}\text{x}}{\text{a}}\text{s}\text{i}\text{n}\frac{\text{n}{\uppi\:}\text{y}}{\text{b}}{\text{e}}^{\text{i}{{\upomega\:}}_{\text{m}\text{n}}\text{t}}$$

whereas $\:{\text{B}}_{\text{m}\text{n}}$ denotes a constant representing the mode shape amplitude; $\:{{\upomega\:}}_{\text{m}\text{n}}$ is the resonant frequency, and $\:\text{i}\:=\sqrt{-1}$.

Using Eq. (32) in (31), the resonant frequency of the plate can be obtained as follows:

33a $$\:{\text{G}}_{11}\left[{\left(\frac{\text{m}{\uppi\:}}{\text{a}}\right)}^{4}+{\left(\frac{\text{n}{\uppi\:}}{\text{b}}\right)}^{4}\right]+2\left({\text{G}}_{12}+2{\text{G}}_{66}\right){\left(\frac{\text{m}{\uppi\:}}{\text{a}}\right)}^{2}{\left(\frac{\text{n}{\uppi\:}}{\text{b}}\right)}^{2}-{\uprho\:}\text{h}{{{\upomega\:}}_{\text{m}\text{n}}}^{2}=0$$

Therefore, the exact solution for obtaining the natural frequency of plates for any value of m and n can be obtained as follows:

33b $$\:{{\upomega\:}}_{\text{m}\text{n}}={\left(\frac{{\uppi\:}}{{\uprho\:}\text{h}}\right)}^{2}\sqrt{{\text{G}}_{11}\left[{\left(\frac{\text{m}}{\text{a}}\right)}^{4}+{\left(\frac{\text{n}}{\text{b}}\right)}^{4}\right]+2\left({\text{G}}_{12}+2{\text{G}}_{66}\right){\left(\frac{\text{m}\text{n}}{\text{a}\text{b}}\right)}^{2}}$$

In this study, we only consider the mode (1,1) resonant frequency $\:{{\upomega\:}}_{11}$ (i.e., fundamental frequency) with a variation in the aspect ratio and thickness of the plate.

The above complete Navier solution is only valid for SSSS rectangular plates subjected to different loading conditions, including UDL, sinusoidal, point, inline, and varying distributed load (VDL). Similar to Navier’s solution, Levy’s theory for rectangular plates (see^64^ for detailed explanation) can be applied for two opposite edges simply supported, and the other two edges are subjected to any arbitrary boundary conditions. Therefore, we also used the approximate Ritz method for arbitrary boundary conditions and validated the Navier solution.

#### Generalized formulation of Ritz method

##### Solution for static bending of rectangular plates considering flexoelectricity

Levy’s theory uses the Fourier series to solve for rectangular plates with two opposite edges simply supported and the other two edges subjected to any arbitrary boundary conditions:

34 $$\:{w}_{0}\left(x,y\right)=\sum\:_{n=1}^{\infty\:}{w}_{0}\left(x\right)\text{sin}{\upbeta\:}\text{y}$$

In this study, we implemented the Ritz method to solve the problem of static bending of rectangular plates. The expression of virtual work (or weak form) and total potential energy for a rectangular plate^55^ can be given as:

35 $$\:0={\int\:}_{0}^{\text{b}}{\int\:}_{0}^{\text{a}}\left[\begin{array}{c}{\text{G}}_{11}\left(\begin{array}{c}\frac{{\partial\:}^{2}{\text{w}}_{0}}{\partial\:{\text{x}}^{2}}\frac{{\partial\:}^{2}{\updelta\:}{\text{w}}_{0}}{\partial\:{\text{x}}^{2}}+\\\:\frac{{\partial\:}^{2}{\text{w}}_{0}}{\partial\:{\text{y}}^{2}}\frac{{\partial\:}^{2}{\updelta\:}{\text{w}}_{0}}{\partial\:{\text{y}}^{2}}\end{array}\right)+4{\text{G}}_{66}\left(\frac{{\partial\:}^{2}{\text{w}}_{0}}{\partial\:\text{x}\partial\:\text{y}}\frac{{\partial\:}^{2}{\updelta\:}{\text{w}}_{0}}{\partial\:\text{y}\partial\:\text{x}}\right)+{\text{G}}_{12}\left(\begin{array}{c}\frac{{\partial\:}^{2}{\text{w}}_{0}}{\partial\:{\text{y}}^{2}}\frac{{\partial\:}^{2}{{\updelta\:}\text{w}}_{0}}{\partial\:{\text{x}}^{2}}+\\\:\frac{{\partial\:}^{2}{\text{w}}_{0}}{\partial\:{\text{x}}^{2}}\frac{{\partial\:}^{2}{{\updelta\:}\text{w}}_{0}}{\partial\:{\text{y}}^{2}}\end{array}\right)\\\:-{\text{q}}_{0}{{\updelta\:}\text{w}}_{0}\end{array}\right]\text{d}\text{x}\text{d}\text{y}$$

Here, the total potential energy functional is given by:

36 $$\:{\Pi\:}\left({\text{w}}_{0}\right)=\frac{1}{2}{\int\:}_{0}^{\text{b}}{\int\:}_{0}^{\text{a}}\left[\begin{array}{c}{\text{G}}_{11}\left(\frac{{\partial\:}^{2}{\text{w}}_{0}}{\partial\:{\text{x}}^{2}}+\frac{{\partial\:}^{2}{\text{w}}_{0}}{\text{d}{\text{y}}^{2}}\right)+4{\text{G}}_{66}\left(\frac{{\partial\:}^{2}{\text{w}}_{0}}{\partial\:\text{y}\partial\:\text{x}}\right)+{2\text{G}}_{12}\left(\frac{{\partial\:}^{2}{\text{w}}_{0}}{\partial\:{\text{y}}^{2}}\frac{{\partial\:}^{2}{\text{w}}_{0}}{\partial\:{\text{x}}^{2}}\right)\\\:-2{q}_{0}{{\updelta\:}\text{w}}_{0}\end{array}\right]\text{d}\text{x}\text{d}\text{y}$$

The general form of the Ritz approximation with N-parameters for the transverse deflection of a plate could be written as:

37 $$\:{\text{w}}_{0}\left(\text{x},\text{y}\right)\approx\:\sum\:_{\text{j}=1}^{\text{N}}{\text{c}}_{\text{j}}{{\upphi\:}}_{\text{j}}\left(\text{x},\text{y}\right)$$

By making use of the above Eqs. (36 and 37) into (35), we obtain:

38 $$\:\left[\text{R}\right]\left\{\text{c}\right\}=\left\{\text{F}\right\}$$

where

39a $$\:{\text{R}}_{\text{i}\text{j}}={\int\:}_{0}^{\text{b}}{\int\:}_{0}^{\text{a}}\left[\begin{array}{c}{\text{G}}_{11}\left(\frac{{\partial\:}^{2}{{\upphi\:}}_{\text{i}}}{\partial\:{\text{x}}^{2}}\frac{{\partial\:}^{2}{{\upphi\:}}_{\text{j}}}{\partial\:{\text{x}}^{2}}+\frac{{\partial\:}^{2}{{\upphi\:}}_{\text{i}}}{\partial\:{\text{y}}^{2}}\frac{{\partial\:}^{2}{{\upphi\:}}_{\text{j}}}{\partial\:{\text{y}}^{2}}\right)+4{\text{G}}_{66}\left(\frac{{\partial\:}^{2}{{\upphi\:}}_{\text{i}}}{\partial\:\text{x}\partial\:\text{y}}\frac{{\partial\:}^{2}{{\upphi\:}}_{\text{j}}}{\partial\:\text{y}\partial\:\text{x}}\right)\\\:+{\text{G}}_{12}\left(\begin{array}{c}\frac{{\partial\:}^{2}{{\upphi\:}}_{\text{i}}}{\partial\:{\text{y}}^{2}}\frac{{\partial\:}^{2}{{\upphi\:}}_{\text{j}}}{\partial\:{\text{x}}^{2}}+\frac{{\partial\:}^{2}{{\upphi\:}}_{\text{i}}}{\partial\:{\text{x}}^{2}}\frac{{\partial\:}^{2}{{\upphi\:}}_{\text{j}}}{\partial\:{\text{y}}^{2}}\end{array}\right)\end{array}\right]\text{d}\text{x}\text{d}\text{y}$$

39b $$\:{\text{F}}_{\text{i}}={\int\:}_{0}^{\text{b}}{\int\:}_{0}^{\text{a}}\left[{q}_{0}{{\upphi\:}}_{\text{i}}\right]\text{d}\text{x}\text{d}\text{y}$$

The general form of the Ritz approximation for the transverse deflection of a plate could be written as:

40 $$\:{\text{w}}_{0}\left(\text{x},\text{y}\right)\approx\:{\text{w}}_{\text{m}\text{n}}\left(\text{x},\text{y}\right)=\sum\:_{\text{i}=1}^{\text{M}}\sum\:_{\text{j}=1}^{\text{N}}{\text{c}}_{\text{i}\text{j}}{{\upphi\:}}_{\text{i}\text{j}}\left(\text{x},\text{y}\right)$$

$\:{\text{c}}_{\text{i}\text{j}}$ are the coefficients to be determined and $\:{{\upphi\:}}_{\text{i}\text{j}}$ represents the trial functions or basis functions that depend on the form of the approximation. In the context of rectangular geometry, it is often convenient to express the approximation function $\:{{\upphi\:}}_{\text{i}\text{j}}(\text{x},\text{y})$ as a tensor product of one-dimensional functions $\:{\text{X}}_{\text{i}}\:$and $\:{\text{Y}}_{\text{j}}$​.

41a $$\:{{\upphi\:}}_{\text{i}\text{j}}\left(\text{x},\text{y}\right)={\text{X}}_{\text{i}}\left(\text{x}\right){\text{Y}}_{\text{j}}\left(\text{y}\right)\:\:\:\text{w}\text{h}\text{e}\text{r}\text{e}\:\text{i}=\text{1,2},\dots\:,\text{m};\text{j}=\text{1,2},\dots\:,\text{n}.$$

From Eqs. (40) and (41a), we have

41b $$\:{\text{w}}_{0}\left(\text{x},\text{y}\right)\approx\:{\text{w}}_{\text{m}\text{n}}\left(\text{x},\text{y}\right)=\sum\:_{\text{i}=1}^{\text{M}}\sum\:_{\text{j}=1}^{\text{N}}{\text{c}}_{\text{i}\text{j}}{\text{X}}_{\text{i}}\left(\text{x}\right){\text{Y}}_{\text{j}}\left(\text{y}\right)$$

In this, boundary conditions associated with the clamped edges are deduced as follows:

At $$\:x=0,\:a:\:\:\:\:\:\:\:{\text{w}}_{0}=0\:\text{a}\text{n}\text{d}\:\frac{\partial\:{\text{w}}_{0}}{\partial\:\text{x}}=0$$ Or

At $$\:y=0,\:b:\:\:\:\:\:\:\:{\text{w}}_{0}=0\:\text{a}\text{n}\text{d}\:\frac{\partial\:{\text{w}}_{0}}{\partial\:\text{y}}=0$$

If the edges of plates are simply supported, it can be deduced as:

$$\:\text{a}\text{t}\:\text{x}\:=0,\:\text{a}:\:{\text{w}}_{0}=0,\:{\text{M}}_{\text{x}\text{x}}=0\:\:\text{O}\text{r}$$

$$\:\text{a}\text{t}\:\text{y}\:=0,\:\text{b}:\:{\text{w}}_{0}=0,\:{\text{M}}_{\text{y}\text{y}}=0.$$

Boundary conditions associated with CCCC, CCSS, and SSSS plates are expressed in Table 1.

**Table 1:**
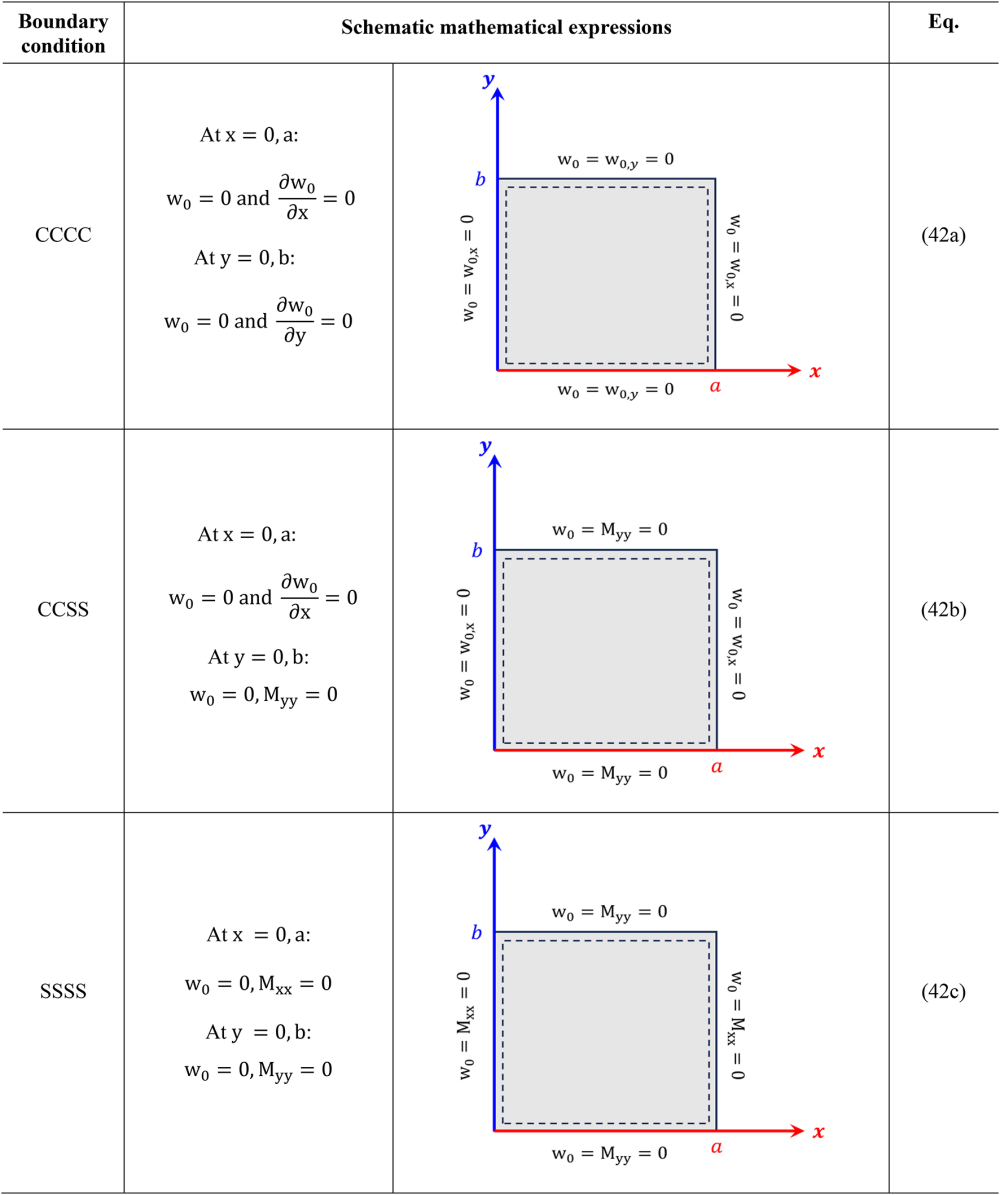
Boundary conditions associated with CCCC, CCSS, and SSSS plates.

Further, $\:\text{M}$ and $\:\text{N}$ may be infinity (i.e., Eq. (34) signifying an infinite series). Thus, the typical approximate functions for $\:{\text{X}}_{\text{i}}$ and $\:{\text{Y}}_{\text{j}}$ for CCCC, CCSS, and SSSS plates were selected from^64^.

43a $$\begin{aligned} {\text{CCCC}}: & \:{\text{X}}_{{\text{i}}} \left( {\text{x}} \right) = \left( {\frac{{\text{x}}}{{\text{a}}}} \right)^{{{\text{i}} + 1}} - 2\left( {\frac{{\text{x}}}{{\text{a}}}} \right)^{{{\text{i}} + 2}} + \left( {\frac{{\text{x}}}{{\text{a}}}} \right)^{{{\text{i}} + 3}} \\ & {\text{Y}}_{{\text{j}}} \left( {\text{y}} \right) = \left( {\frac{{\text{y}}}{{\text{b}}}} \right)^{{{\text{j}} + 1}} - 2\left( {\frac{{\text{y}}}{{\text{b}}}} \right)^{{{\text{j}} + 2}} + \left( {\frac{{\text{y}}}{{\text{b}}}} \right)^{{{\text{j}} + 3}} \\ \end{aligned}$$

43b $$\begin{aligned} {\text{CCSS}}: & \:{\text{X}}_{{\text{i}}} \left( {\text{x}} \right) = \left( {\frac{{\text{x}}}{{\text{a}}}} \right)^{{{\text{i}} + 1}} - 2\left( {\frac{{\text{x}}}{{\text{a}}}} \right)^{{{\text{i}} + 2}} + \left( {\frac{{\text{x}}}{{\text{a}}}} \right)^{{{\text{i}} + 3}} \\ & {\text{Y}}_{{\text{j}}} \left( {\text{y}} \right) = \left( {\frac{{\text{y}}}{{\text{b}}}} \right)^{{\text{j}}} - \left( {\frac{{\text{y}}}{{\text{b}}}} \right)^{{{\text{j}} + 1}} \\ \end{aligned}$$

43c $$\begin{aligned} {\text{SSSS}}: & {\text{X}}_{{\text{i}}} \left( {\text{x}} \right) = \left( {\frac{{\text{x}}}{{\text{a}}}} \right)^{{\text{i}}} - \left( {\frac{{\text{x}}}{{\text{a}}}} \right)^{{{\text{i}} + 1}} \\ & {\text{Y}}_{{\text{j}}} \left( {\text{y}} \right) = \left( {\frac{{\text{y}}}{{\text{b}}}} \right)^{{\text{j}}} - \left( {\frac{{\text{y}}}{{\text{b}}}} \right)^{{{\text{j}} + 1}} \\ \end{aligned}$$

where $$\:,\:\text{i}=\text{1,2},\dots\:,\text{m},\:\text{a}\text{n}\text{d}\:\text{j}=\text{1,2},\dots\:,\text{n}$$

###### Application of Ritz method: static solution for CCCC plate

When considering a composite plate with all edges clamped and subjected to a distributed transverse load, $\:{q}_{0}(x,\:y)$, the boundary conditions associated with a clamped plate typically include constraints on the plate’s displacement and rotation at each edge. For CCCC plates, the boundary conditions and the approximate functions in Eqs. (42a and 43a) can be used.

Therefore, by making use of Eq. (40) with $\:{{\upphi\:}}_{\text{i}\text{j}}$ given by Eq. (41a) and $\:{{\updelta\:}\text{W}}_{0}=\sum\:_{\text{p}}^{\text{m}}\sum\:_{\text{q}}^{\text{n}}{{\updelta\:}\text{c}}_{\text{p}\text{q}}{{\upphi\:}}_{\text{p}\text{q}}$ into Eq. (35), we get^55^

44a $$\:0=\sum\:_{\text{p}}^{\text{m}}\sum\:_{\text{q}}^{\text{n}}\left\{\sum\:_{\text{i}}^{\text{m}}\sum\:_{\text{j}}^{\text{n}}{\text{c}}_{\text{i}\text{j}}{\int\:}_{0}^{\text{b}}{\int\:}_{0}^{\text{a}}\left[\begin{array}{c}{\text{G}}_{11}\left(\frac{{\text{d}}^{2}{\text{X}}_{\text{i}}}{\text{d}{\text{x}}^{2}}{\text{Y}}_{\text{j}}\frac{{\text{d}}^{2}{\text{X}}_{\text{p}}}{\text{d}{\text{x}}^{2}}{\text{Y}}_{\text{q}}\right)+\\\:4{\text{G}}_{66}\left(\frac{\text{d}{\text{X}}_{\text{i}}}{\text{d}\text{x}}\frac{\text{d}{\text{Y}}_{\text{j}}}{\text{d}\text{y}}\frac{\text{d}{\text{X}}_{\text{p}}}{\text{d}\text{x}}\frac{\text{d}{\text{Y}}_{\text{q}}}{\text{d}\text{y}}\right)+\\\:{\text{G}}_{12}\left(\begin{array}{c}{\text{X}}_{\text{i}}\frac{{\text{d}}^{2}{\text{Y}}_{\text{j}}}{\text{d}{\text{y}}^{2}}\frac{{\text{d}}^{2}{\text{X}}_{\text{p}}}{\text{d}{\text{x}}^{2}}{\text{Y}}_{\text{q}}+\\\:\frac{{\text{d}}^{2}{\text{X}}_{\text{i}}}{\text{d}{\text{x}}^{2}}{\text{Y}}_{\text{j}}{\text{X}}_{\text{p}}\frac{{\text{d}}^{2}{\text{Y}}_{\text{q}}}{\text{d}{\text{y}}^{2}}\end{array}\right)\end{array}\right]\text{d}\text{x}\text{d}\text{y}-{\int\:}_{0}^{\text{b}}{\int\:}_{0}^{\text{a}}{q}_{0}{\text{X}}_{\text{p}}{\text{Y}}_{\text{q}}\text{d}\text{x}\text{d}\text{y}\right\}{{\updelta\:}\text{c}}_{\text{p}\text{q}}$$

Meanwhile, the statement should hold for any arbitrary variations $\:{{\updelta\:}\text{c}}_{\text{p}\text{q}}$, the expression inside the curly bracket should be zero for all $\:\text{p},\:\text{q}=\text{1,2},\dots\::$

44b $$\:0=\sum\:_{\text{i}}^{\text{m}}\sum\:_{\text{j}}^{\text{n}}\left\{{\int\:}_{0}^{\text{b}}{\int\:}_{0}^{\text{a}}\left[\begin{array}{c}{\text{G}}_{11}\left(\frac{{\text{d}}^{2}{\text{X}}_{\text{i}}}{\text{d}{\text{x}}^{2}}{\text{Y}}_{\text{j}}\frac{{\text{d}}^{2}{\text{X}}_{\text{p}}}{\text{d}{\text{x}}^{2}}{\text{Y}}_{\text{q}}\right)+\\\:4{\text{G}}_{66}\left(\frac{\text{d}{\text{X}}_{\text{i}}}{\text{d}\text{x}}\frac{\text{d}{\text{Y}}_{\text{j}}}{\text{d}\text{y}}\frac{\text{d}{\text{X}}_{\text{p}}}{\text{d}\text{x}}\frac{\text{d}{\text{Y}}_{\text{q}}}{\text{d}\text{y}}\right)+\\\:{\text{G}}_{12}\left(\begin{array}{c}{\text{X}}_{\text{i}}\frac{{\text{d}}^{2}{\text{Y}}_{\text{j}}}{\text{d}{\text{y}}^{2}}\frac{{\text{d}}^{2}{\text{X}}_{\text{p}}}{\text{d}{\text{x}}^{2}}{\text{Y}}_{\text{q}}+\\\:\frac{{\text{d}}^{2}{\text{X}}_{\text{i}}}{\text{d}{\text{x}}^{2}}{\text{Y}}_{\text{j}}{\text{X}}_{\text{p}}\frac{{\text{d}}^{2}{\text{Y}}_{\text{q}}}{\text{d}{\text{y}}^{2}}\end{array}\right)\end{array}\right]\text{d}\text{x}\text{d}\text{y}\right\}{\text{c}}_{\text{i}\text{j}}-{\int\:}_{0}^{\text{b}}{\int\:}_{0}^{\text{a}}{q}_{0}{\text{X}}_{\text{p}}{\text{Y}}_{\text{q}}\text{d}\text{x}\text{d}\text{y}$$

The above expression indicates $\:\text{m}\times\:\text{n}$ algebraic equations among the coefficients $\:{\text{c}}_{\text{i}\text{j}}$. Note that all integrals in the above expressions are line integrals, and they are involved in estimating these integrals:

45a $$\:{\int\:}_{0}^{\text{a}}{\text{X}}_{\text{i}}\:\text{d}\text{x},\:{\int\:}_{0}^{\text{a}}{\text{X}}_{\text{i}}{\text{X}}_{\text{p}}\:\text{d}\text{x},\:{\int\:}_{0}^{\text{a}}\frac{\text{d}{\text{X}}_{\text{i}}}{\text{d}\text{x}}\frac{\text{d}{\text{X}}_{\text{p}}}{\text{d}\text{x}}\:\text{d}\text{x},\:{\int\:}_{0}^{\text{a}}{\text{X}}_{\text{i}}\frac{{\text{d}}^{2}{\text{X}}_{\text{p}}}{\text{d}{\text{x}}^{2}}\:\text{d}\text{x},\:{\int\:}_{0}^{\text{a}}\frac{{\text{d}}^{2}{\text{X}}_{\text{i}}}{\text{d}{\text{x}}^{2}}\:\frac{{\text{d}}^{2}{\text{X}}_{\text{p}}}{\text{d}{\text{x}}^{2}}\text{d}\text{x}$$

For this, if we consider $\:\text{m}=\text{n}=1$, we get:

45b $$\begin{gathered} \int\limits_{0}^{{\text{a}}} {{\text{X}}_{1} \:{\text{dx}}} = \frac{{\text{a}}}{{30}},\int\limits_{0}^{{\text{a}}} {{\text{X}}_{1} {\text{X}}_{1} \:{\text{dx}}} = \frac{{\text{a}}}{{630}},\int\limits_{0}^{{\text{a}}} {\frac{{{\text{dX}}_{1} }}{{{\text{dx}}}}\frac{{{\text{dX}}_{1} }}{{{\text{dx}}}}\:{\text{dx}}} = \frac{2}{{105{\text{a}}}}, \hfill \\ \int\limits_{0}^{{\text{a}}} {{\text{X}}_{1} \frac{{{\text{d}}^{2} {\text{X}}_{1} }}{{{\text{dx}}^{2} }}\:{\text{dx}}} = - \frac{2}{{105{\text{a}}}},\int\limits_{0}^{{\text{a}}} {\frac{{{\text{d}}^{2} {\text{X}}_{1} }}{{{\text{dx}}^{2} }}\:\frac{{{\text{d}}^{2} {\text{X}}_{1} }}{{{\text{dx}}^{2} }}{\text{dx}}} = \frac{4}{{5{\text{a}}^{3} }}, \hfill \\ \end{gathered}$$

By putting the integral values into Eq. (44b). we get:

46a $$\:0=\left[\begin{array}{c}{\left(\frac{4}{{5\text{a}}^{3}}\right)\left(\frac{\text{b}}{630}\right)\text{G}}_{11}+4{\text{G}}_{66}\left(\frac{2}{105\text{a}}\right)\left(\frac{2}{105\text{b}}\right)\\\:+2{\text{G}}_{12}\left(-\frac{2}{105\text{a}}\right)\left(-\frac{2}{105\text{b}}\right)\end{array}\right]{\text{c}}_{11}-\left(\frac{\text{a}\text{b}}{900}\right){\text{q}}_{0}$$

And the one-parameter Ritz solution for CCCC plate becomes:

46b $$\:{\text{W}}_{11}\left(\text{x},\text{y}\right)=\left(\frac{49}{8}\right)\frac{{\text{q}}_{0}{a}^{4}{\left[{\left(\frac{\text{x}}{\text{a}}\right)-\left(\frac{\text{x}}{\text{a}}\right)}^{2}\right]}^{2}{\left[{\left(\frac{\text{y}}{\text{b}}\right)-\left(\frac{\text{y}}{\text{b}}\right)}^{2}\right]}^{2}}{7{\text{G}}_{11}+4\left({\text{G}}_{12}+2{\text{G}}_{66}\right){\text{s}}^{2}}\:\:\text{a}\text{n}\text{d}\:\:\text{s}=\text{a}/\text{b}$$

In case of CCCC plates under UDL loading, the maximum deflection occurs as $\:x=a/2$ and $\:y=b/2$.

##### Solution for dynamic analysis—natural frequency of rectangular plates considering flexoelectricity

For solving problems associated with the natural vibration or frequency of rectangular plates, considering inertia terms ($\:{\text{I}}_{0}={\uprho\:}\text{h}\:\text{a}\text{n}\text{d}\:{\text{I}}_{2}=\frac{{{\uprho\:}\text{h}}^{3}}{12}$ = rotary inertia), Eq. (35) can be rewritten as:

47 $$\:0={\int\:}_{0}^{\text{b}}{\int\:}_{0}^{\text{a}}\left[\begin{array}{c}{\text{G}}_{11}\left(\frac{{\partial\:}^{2}{\text{w}}_{0}}{\partial\:{\text{x}}^{2}}\frac{{\partial\:}^{2}{\updelta\:}{\text{w}}_{0}}{\partial\:{\text{x}}^{2}}+\frac{{\partial\:}^{2}{\text{w}}_{0}}{\partial\:{\text{y}}^{2}}\frac{{\partial\:}^{2}{\updelta\:}{\text{w}}_{0}}{\partial\:{\text{y}}^{2}}\right)+4{\text{G}}_{66}\left(\frac{{\partial\:}^{2}{\text{w}}_{0}}{\partial\:\text{x}\partial\:\text{y}}\frac{{\partial\:}^{2}{\updelta\:}{\text{w}}_{0}}{\partial\:\text{y}\partial\:\text{x}}\right)\\\:+{\text{G}}_{12}\left(\begin{array}{c}\frac{{\partial\:}^{2}{\text{w}}_{0}}{\partial\:{\text{y}}^{2}}\frac{{\partial\:}^{2}{{\updelta\:}\text{w}}_{0}}{\partial\:{\text{x}}^{2}}+\\\:\frac{{\partial\:}^{2}{\text{w}}_{0}}{\partial\:{\text{x}}^{2}}\frac{{\partial\:}^{2}{{\updelta\:}\text{w}}_{0}}{\partial\:{\text{y}}^{2}}\end{array}\right)\\\:-{{\upomega\:}}^{2}\left\{{\text{I}}_{0}{\text{w}}_{0}{\updelta\:}{\text{w}}_{0}+{\text{I}}_{2}\left(\frac{\partial\:{\text{w}}_{0}}{\partial\:\text{x}}\frac{\partial\:{\updelta\:}{\text{w}}_{0}}{\partial\:\text{x}}+\frac{\partial\:{\text{w}}_{0}}{\partial\:\text{y}}\frac{\partial\:{\updelta\:}{\text{w}}_{0}}{\partial\:\text{y}}\right)\right\}\end{array}\right]\text{d}\text{x}\text{d}\text{y}$$

By making use of the above Eqs. (37 and 47), we obtain:

48 $$\:\left(\left[\text{R}\right]-{{\upomega\:}}^{2}\left[\text{B}\right]\right)\left\{\text{c}\right\}=0$$

where,

49a $$\:{\text{R}}_{\text{i}\text{j}}={\int\:}_{0}^{\text{b}}{\int\:}_{0}^{\text{a}}\left[\begin{array}{c}{\text{G}}_{11}\left(\begin{array}{c}\frac{{\partial\:}^{2}{{\upphi\:}}_{\text{i}}}{\partial\:{\text{x}}^{2}}\frac{{\partial\:}^{2}{{\upphi\:}}_{\text{j}}}{\partial\:{\text{x}}^{2}}+\\\:\frac{{\partial\:}^{2}{{\upphi\:}}_{\text{i}}}{\partial\:{\text{y}}^{2}}\frac{{\partial\:}^{2}{{\upphi\:}}_{\text{j}}}{\partial\:{\text{y}}^{2}}\end{array}\right)+4{\text{G}}_{66}\left(\frac{{\partial\:}^{2}{{\upphi\:}}_{\text{i}}}{\partial\:\text{x}\partial\:\text{y}}\frac{{\text{d}}^{2}{{\upphi\:}}_{\text{j}}}{\partial\:\text{y}\partial\:\text{x}}\right)+{\text{G}}_{12}\left(\begin{array}{c}\frac{{\partial\:}^{2}{{\upphi\:}}_{\text{i}}}{\partial\:{\text{y}}^{2}}\frac{{\partial\:}^{2}{{\upphi\:}}_{\text{j}}}{\partial\:{\text{x}}^{2}}+\\\:\frac{{\partial\:}^{2}{{\upphi\:}}_{\text{i}}}{\partial\:{\text{x}}^{2}}\frac{{\partial\:}^{2}{{\upphi\:}}_{\text{j}}}{\partial\:{\text{y}}^{2}}\end{array}\right)\end{array}\right]\text{d}\text{x}\text{d}\text{y}$$

49b $$\:{\text{B}}_{\text{i}\text{j}}={\int\:}_{0}^{\text{b}}{\int\:}_{0}^{\text{a}}\left[\left\{{\text{I}}_{0}{{\upphi\:}}_{\text{i}}{{\upphi\:}}_{\text{i}}+{\text{I}}_{2}\left(\frac{\partial\:{{\upphi\:}}_{\text{i}}}{\partial\:\text{x}}\frac{\partial\:{{\upphi\:}}_{\text{i}}}{\partial\:\text{x}}+\frac{\partial\:{{\upphi\:}}_{\text{i}}}{\partial\:\text{y}}\frac{\partial\:{{\upphi\:}}_{\text{i}}}{\partial\:\text{y}}\right)\right\}\right]\text{d}\text{x}\text{d}\text{y}$$

From Eqs. (40, 41, 43, 47 and 48), the coefficient of $\:{\text{R}}_{\left(\text{i}\text{j}\right),\left(\text{k}\text{l}\right)}\:\text{a}\text{n}\text{d}\:{\text{B}}_{\left(\text{i}\text{j}\right),\left(\text{k}\text{l}\right)}$ of $\:\left[\text{R}\right]\:\text{a}\text{n}\text{d}\:\left[\text{B}\right]$ are defined as:

50a $$\:{\text{R}}_{\text{i}\text{j},\text{k}\text{l}}={\int\:}_{0}^{\text{b}}{\int\:}_{0}^{\text{a}}\left[\begin{array}{c}{\text{G}}_{11}\left(\frac{{\text{d}}^{2}{\text{X}}_{\text{i}}}{\text{d}{\text{x}}^{2}}\frac{{\text{d}}^{2}{\text{X}}_{\text{k}}}{\text{d}{\text{x}}^{2}}{\text{Y}}_{\text{j}}{\text{Y}}_{\text{l}}+\frac{{\text{d}}^{2}{\text{Y}}_{\text{i}}}{\text{d}{\text{y}}^{2}}\frac{{\text{d}}^{2}{\text{X}\text{Y}}_{\text{k}}}{\text{d}{\text{y}}^{2}}{\text{X}}_{\text{i}}{\text{X}}_{\text{k}}\right)+\\\:{\text{G}}_{12}\left(\begin{array}{c}{\text{X}}_{\text{i}}\frac{{\text{d}}^{2}{\text{X}}_{\text{k}}}{\text{d}{\text{x}}^{2}}\frac{{\text{d}}^{2}{\text{Y}}_{\text{j}}}{\text{d}{\text{y}}^{2}}{\text{Y}}_{\text{l}}+\\\:\frac{{\text{d}}^{2}{\text{X}}_{\text{i}}}{\text{d}{\text{x}}^{2}}{\text{X}}_{\text{k}}{\text{Y}}_{\text{j}}\frac{{\text{d}}^{2}{\text{Y}}_{\text{l}}}{\text{d}{\text{y}}^{2}}\end{array}\right)+4{\text{G}}_{66}\left(\frac{\text{d}{\text{X}}_{\text{i}}}{\text{d}\text{x}}\frac{\text{d}{\text{X}}_{\text{k}}}{\text{d}\text{x}}\frac{\text{d}{\text{Y}}_{\text{j}}}{\text{d}\text{y}}\frac{\text{d}{\text{Y}}_{\text{l}}}{\text{d}\text{y}}\right)\end{array}\right]\text{d}\text{x}\text{d}\text{y}$$

50b $$\:{\text{B}}_{\text{i}\text{j},\text{k}\text{l}}={\int\:}_{0}^{\text{b}}{\int\:}_{0}^{\text{a}}\left[\left\{{\text{I}}_{0}{\text{X}}_{\text{i}}{\text{X}}_{\text{k}}{\text{Y}}_{\text{j}}{\text{Y}}_{\text{l}}+{\text{I}}_{2}\left(\frac{\text{d}{\text{X}}_{\text{i}}}{\text{d}\text{x}}\frac{\text{d}{\text{X}}_{\text{k}}}{\text{d}\text{x}}{\text{Y}}_{\text{j}}{\text{Y}}_{\text{l}}+{\text{X}}_{\text{i}}{\text{X}}_{\text{k}}\frac{\text{d}{\text{Y}}_{\text{j}}}{\text{d}\text{y}}\frac{\text{d}{\text{Y}}_{\text{l}}}{\text{d}\text{y}}\right)\right\}\right]\text{d}\text{x}\text{d}\text{y}$$

Here, by neglecting rotary inertia ($\:{\text{I}}_{2}$) as it is negligible, the above Eq. (50b) becomes:

50c $$\:{\text{B}}_{\text{i}\text{j},\text{k}\text{l}}={\int\:}_{0}^{\text{b}}{\int\:}_{0}^{\text{a}}\left[{\text{I}}_{0}{\text{X}}_{\text{i}}{\text{X}}_{\text{k}}{\text{Y}}_{\text{j}}{\text{Y}}_{\text{l}}\right]\text{d}\text{x}\text{d}\text{y}$$

From the above Eq. (48), one can obtain the natural frequency of rectangular plates with all edge support conditions. In this study, we neglected the in-plane vibration of plates. Again, the general form of the Ritz approximation for the natural frequency of rectangular plates could be rewritten as:

51 $$\:{\text{w}}_{0}\left(\text{x},\text{y}\right)\approx\:{\text{w}}_{\text{m}\text{n}}\left(\text{x},\text{y}\right)=\sum\:_{\text{i}=1}^{\text{M}}\sum\:_{\text{j}=1}^{\text{N}}{\text{c}}_{\text{i}\text{j}}{{\upphi\:}}_{\text{i}\text{j}}\left(\text{x},\text{y}\right)=\sum\:_{\text{i}=1}^{\text{M}}\sum\:_{\text{j}=1}^{\text{N}}{\text{c}}_{\text{i}\text{j}}{\text{X}}_{\text{i}}\left(\text{x}\right){\text{Y}}_{\text{j}}\left(\text{y}\right)$$

The size of the matrix $\:\left[\text{R}\right]$ is $\:\text{M}\text{N}\times\:\text{N}\text{M},$ and the Eq. (48) can be used to determine the first $\:\text{M}\text{N}$ frequencies of the infinite set. From this, it is clear that the natural frequencies ($\:{{\upomega\:}}_{\text{m}\text{n}}$) can be obtained for any values of $\:\text{m}$ and $\:\text{n}$. The Ritz method gives accurate results for natural frequencies if one chooses a larger value of M and N.

###### Application of Ritz method: dynamic solution for SSSS plate

Using Eqs. (43c) and (51), the Ritz approximation for MGHPC plates can be written as follows:

52 $$\:{\text{w}}_{0}\left(\text{x},\text{y}\right)=\sum\:_{\text{m}=1}^{\text{M}}\sum\:_{\text{n}=1}^{\text{N}}{\text{c}}_{\text{m}\text{n}}\left[{\left(\:\frac{\text{x}}{\text{a}}\:\right)}^{\text{m}}-{\left(\:\frac{\text{x}}{\text{a}}\:\right)}^{\text{m}+1}\right]\left[{\left(\:\frac{\text{y}}{\text{b}}\:\right)}^{\text{n}}-{\left(\:\frac{\text{y}}{\text{b}}\:\right)}^{\text{n}+1}\right]$$

Derivatives of the above equation can be expressed as:

53a $$\:\frac{\partial\:{\text{w}}_{0}}{\partial\:\text{x}}=\sum\:_{\text{m}=1}^{\text{M}}\sum\:_{\text{n}=1}^{\text{N}}{\text{c}}_{\text{m}\text{n}}\left(\frac{\text{m}}{\text{a}}{\left(\:\frac{\text{x}}{\text{a}}\:\right)}^{\text{m}-1}-\frac{\text{m}+1}{\text{a}}{\left(\:\frac{\text{x}}{\text{a}}\:\right)}^{\text{m}}\right)\left[{\left(\:\frac{\text{y}}{\text{b}}\:\right)}^{\text{n}}-{\left(\:\frac{\text{y}}{\text{b}}\:\right)}^{\text{n}+1}\right]$$

53b $$\:\frac{{\partial\:}^{2}{\text{w}}_{0}}{\partial\:{\text{x}}^{2}}=\sum\:_{\text{m}=1}^{\text{M}}\sum\:_{\text{n}=1}^{\text{N}}{\text{c}}_{\text{m}\text{n}}\left(\frac{\text{m}(\text{m}-1)}{{\text{a}}^{2}}{\left(\:\frac{\text{x}}{\text{a}}\:\right)}^{\text{m}-2}\right)\left[{\left(\:\frac{\text{y}}{\text{b}}\:\right)}^{\text{n}}-{\left(\:\frac{\text{y}}{\text{b}}\:\right)}^{\text{n}+1}\right]$$

Similarly, we can get $\:\frac{\partial\:{\text{w}}_{0}}{\partial\:\text{y}},\:\frac{{\partial\:}^{2}{\text{w}}_{0}}{\partial\:{\text{y}}^{2}}\:\text{a}\text{n}\text{d}\:\frac{{\partial\:}^{2}{\text{w}}_{0}}{\partial\:\text{x}\partial\:\text{y}}.$

53c $$\:\frac{\partial\:{\text{w}}_{0}}{\partial\:\text{y}}=\sum\:_{\text{m}=1}^{\text{M}}\sum\:_{\text{n}=1}^{\text{N}}{\text{c}}_{\text{m}\text{n}}\left(\frac{\text{n}}{\text{b}}{\left(\:\frac{\text{y}}{\text{b}}\:\right)}^{\text{n}-1}-\frac{\text{n}+1}{\text{b}}{\left(\:\frac{\text{y}}{\text{b}}\:\right)}^{\text{n}}\right)\left[{\left(\:\frac{\text{x}}{\text{a}}\:\right)}^{\text{m}}-{\left(\:\frac{\text{x}}{\text{a}}\:\right)}^{\text{m}+1}\right]$$

53d $$\:\frac{{\partial\:}^{2}{\text{w}}_{0}}{\partial\:{\text{y}}^{2}}=\sum\:_{\text{m}=1}^{\text{M}}\sum\:_{\text{n}=1}^{\text{N}}{\text{c}}_{\text{m}\text{n}}\left(\frac{\text{n}(\text{n}-1)}{{\text{b}}^{2}}{\left(\:\frac{\text{y}}{\text{b}}\:\right)}^{\text{n}-2}\right)\left[{\left(\:\frac{\text{x}}{\text{a}}\:\right)}^{\text{m}}-{\left(\:\frac{\text{x}}{\text{a}}\:\right)}^{\text{m}+1}\right]\:\:\:\:\text{a}\text{n}\text{d}$$

53e $$\:\frac{{\partial\:}^{2}{\text{w}}_{0}}{\partial\:\text{x}\partial\:\text{y}}=\sum\:_{\text{m}=1}^{\text{M}}\sum\:_{\text{n}=1}^{\text{N}}{\text{c}}_{\text{m}\text{n}}\frac{\text{m}}{\text{a}}{\frac{\text{n}}{\text{b}}\left(\:\frac{\text{x}}{\text{a}}\:\right)}^{\text{m}-1}{\left(\:\frac{\text{y}}{\text{b}}\:\right)}^{\text{n}-1}$$

Now, we can minimize the total potential energy (Eq. 36) with respect to the unknown $\:{\text{c}}_{\text{m}\text{n}}$ coefficient and set it to zero.

54 $$\:\frac{\partial\:{\Pi\:}}{\partial\:{\text{c}}_{\text{m}\text{n}}}=0$$

The minimization process in the Ritz leads to an eigenvalue problem where the eigenvalues correspond to the natural frequencies $\:{{{\upomega\:}}_{\text{m}\text{n}}}^{2}$. This problem takes the general form presented in Eq. (48).

After performing the necessary integrations and solving the eigenvalue problem, the natural frequencies $\:{{\upomega\:}}_{\text{m}\text{n}}$ for SSSS plates can be expressed as:

55 $$\:{{{\upomega\:}}_{\text{m}\text{n}}}^{2}=\frac{{{\uppi\:}}^{4}}{{\uprho\:}\text{h}}\left(\frac{{\text{G}}_{11}{\text{m}}^{4}}{{\text{a}}^{4}}+\frac{{\text{G}}_{11}{\text{n}}^{4}}{{\text{b}}^{4}}+\frac{{2\text{G}}_{12}{\text{m}}^{2}{\text{n}}^{2}}{{\text{a}}^{2}{\text{b}}^{2}}+\frac{{4\text{G}}_{66}{\text{m}}^{2}{\text{n}}^{2}}{{\text{a}}^{2}{\text{b}}^{2}}\right)$$

If one compares the final solution using Ritz and Navier for the SSSS plate, the two solutions for natural frequencies almost exactly coincide for any value of m and n.

## Results and discussion

The derived models were used to examine a comprehensive quantitative analysis concerning the electromechanical behavior of MGHPC plates under various edge support and loading conditions. The mechanical properties of the materials are listed in Table 2.

**Table 2:** Mechanical properties of MXene ($\:{\text{T}\text{i}}_{3}{\text{C}}_{2}{\text{T}}_{x}$), graphene, and epoxy^13,49,65^.

Material properties	Nano-reinforcements		Matrix
	MXene	Graphene	Epoxy
Young’s modulus (GPa)	330	985	2.85
Poisson’s ratio	0.23	0.265	0.3
Piezoelectric constant (C/m^2^)	–	− 0.227	–

### Effective properties of MXene/graphene-based hybrid piezocomposites

First, the effective properties of MGHPC were determined using a three-phase micromechanical, i.e., mechanics of materials (MOM) model, for two nano-reinforcements, including 2D MXene/graphene and epoxy matrix. The results predicted by MOM models were validated using previously reported experimental data^49^ for 0.112% volume fraction (0.1% weight fraction). It is observed that the results obtained from the MOM (3.865 GPa) for graphene-reinforced composites are in better agreement with experimental results (3.740 GPa)^49^.

**Fig. 4:**
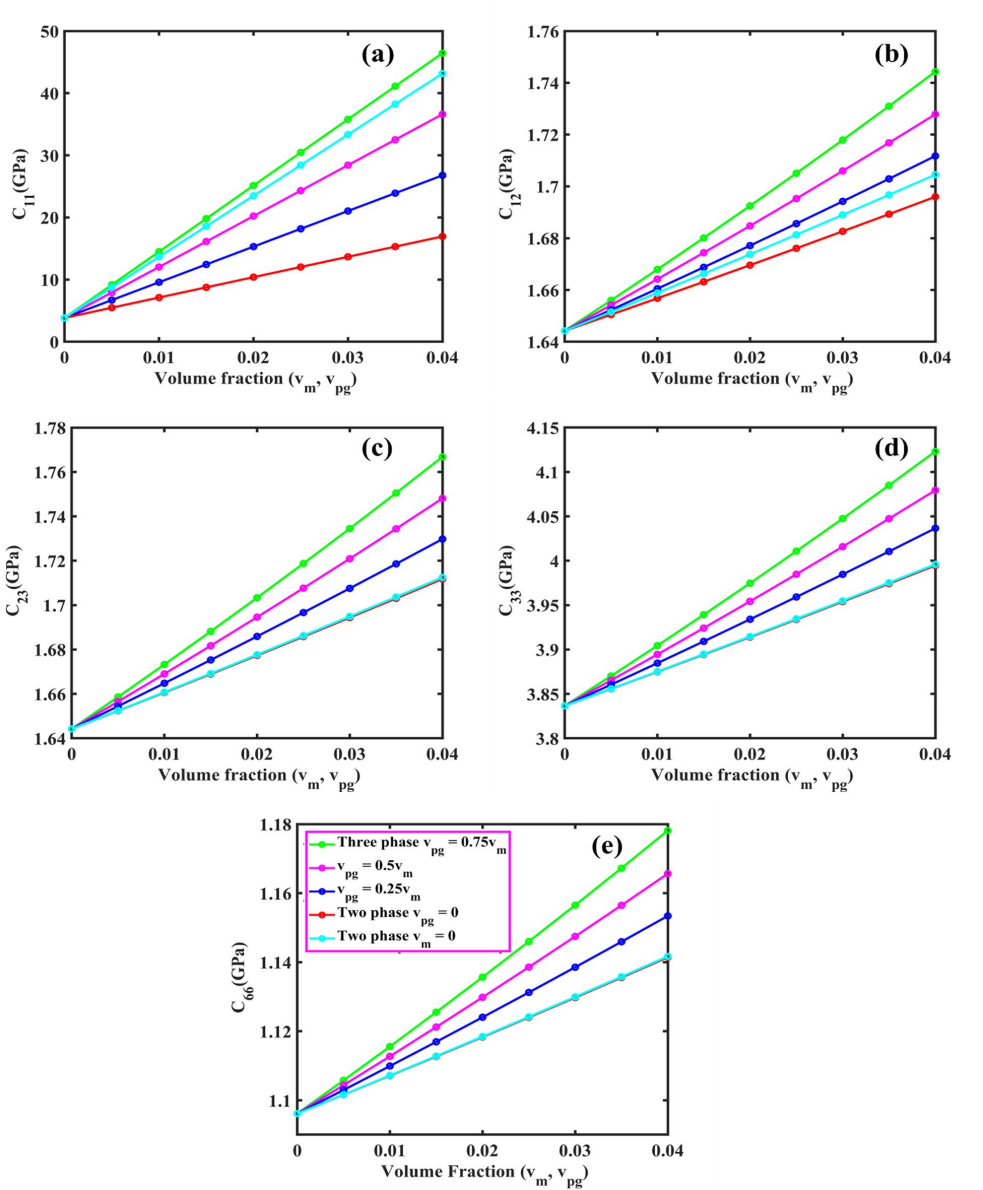
Comparison of effective elastic properties (**a**) $\:{\text{C}}_{11}$, (**b**) $\:{\text{C}}_{12}$, (**c**) $\:{\text{C}}_{23}$, (**d**) $\:{\text{C}}_{33},\:$and (**e**) $\:{\text{C}}_{66}$ of MGHPC for different choices of $\:{\text{v}}_{\text{p}\text{g}}$ and $\:{\text{v}}_{\text{m}}$.

Here, we considered the volume fraction of MXene nano-reinforcements ($\:{\text{v}}_{\text{m}}$) to be constant at 4% and varied volume fraction of graphene nano-reinforcements ($\:{\text{v}}_{\text{p}\text{g}}$ = 1 – 3%) to examine their impact on the effective properties of MGHPC. In addition, we also considered different $\:{\text{v}}_{\text{p}\text{g}}$ values, i.e., 0.75 $\:{\text{v}}_{\text{m}}$, 0.50 $\:{\text{v}}_{\text{m}}$, and 0.25 $\:{\text{v}}_{\text{m}}$ for the three-phase MGHPC. In contrast, the addition of graphene nanofillers was not taken into consideration in MXene-reinforced composites (MRC), while the addition of MXene nanofillers was not considered in graphene-reinforced composites (GRC). Moreover, the hybrid three-phase composite incorporates graphene and MXene nano-reinforcements, which are oriented and poled along the length of the laminate. Furthermore, variations in the composition were considered, including configurations with and without graphene/MXene nano-reinforcements. The effective piezoelastic properties of the MGHPC $\:({\text{C}}_{11}$, $\:{\text{C}}_{12}$, $\:{\text{C}}_{13}$, $\:{\text{C}}_{23}$, $\:{\text{C}}_{33}$, $\:{\text{C}}_{66}$, $\:{\text{e}}_{31}$ and $\:{\text{e}}_{33})$, were determined by accounting for iso-stress/strain conditions and the rule of mixtures assumption. Utilizing Eq. (6), all the effective piezoelastic constants can be calculated with distinct values of $\:{\text{v}}_{\text{p}\text{g}}$ and $\:{\text{v}}_{\text{m}}$. The variation of $\:{\text{C}}_{11}$ concerning the $\:{\text{v}}_{\text{p}\text{g}}$ and $\:{\text{v}}_{\text{m}}\:$is illustrated for both two- and three-phase composites (i.e., MRC, GRC, and MGHPC; Fig. 4a). The values of $\:{\text{C}}_{11}$ exhibit a linear trend concerning $\:{\text{v}}_{\text{p}\text{g}}$ and $\:{\text{v}}_{\text{m}}$. This linear variation is attributed to the adherence to iso-stress/strain assumptions (Eq. 2) and aligns closely with the Voigt upper bound as well as with experimental results mentioned earlier for a 0.112% composition. Notably, incorporating graphene in MGHPC leads to an improved value of $\:{\text{C}}_{11}$ compared to the two-phase MRC ($\:{\text{v}}_{\text{p}\text{g}}=0$), as seen in Fig. 4a. We also validated these axial effective elastic properties of MGHPC using Mori-Tanaka (MT) and FE methods (Table 3). We found that the present MOM model agrees with the MT and FE methods. However, results obtained through MT and FE methods are not presented here. Figure 4(b–d) provide a comparison of results for transverse elastic constants ($\:{\text{C}}_{12}$, $\:{\text{C}}_{13}$, $\:{\text{C}}_{23}$, $\:{\text{C}}_{33}$) of MRC, GRC, and MGHPC. Unlike the linear variation observed for $\:{\text{C}}_{11}$, these results do not exhibit a linear trend because the transverse elastic constants of proposed composites are primarily matrix-dependent. The extension-extension coupling exhibits transverse elastic constants, representing the Poisson’s effect, among distinct normal stresses and strains resulting from the applied load in the reinforcement or 1–direction. The Poisson’s effect refers to the phenomenon where a material contracts or expands in directions perpendicular to the applied load. The values of $\:{\text{C}}_{22}$ and $\:{\text{C}}_{33}$ are almost identical as the axis of symmetry considered in the 1-direction.

**Table 3:** Comparison results for effective elastic properties of MGHPC with $\:{\text{v}}_{\text{p}\text{g}}=5\text{\%}$ and $\:{\text{v}}_{\text{m}}=5\text{\%}$ using MOM and FE modeling.

Effective elastic properties	MOM (GPa)	FE (GPa)
$\:{\text{C}}_{11}$	69.372	69.01
$\:{\text{C}}_{12}$	1.793	1.771
$\:{\text{C}}_{13}$	1.852	1.85
$\:{\text{C}}_{33}$	4.259	4.156
$\:{\text{C}}_{44}$	1.217	1.199

**Fig. 5:**
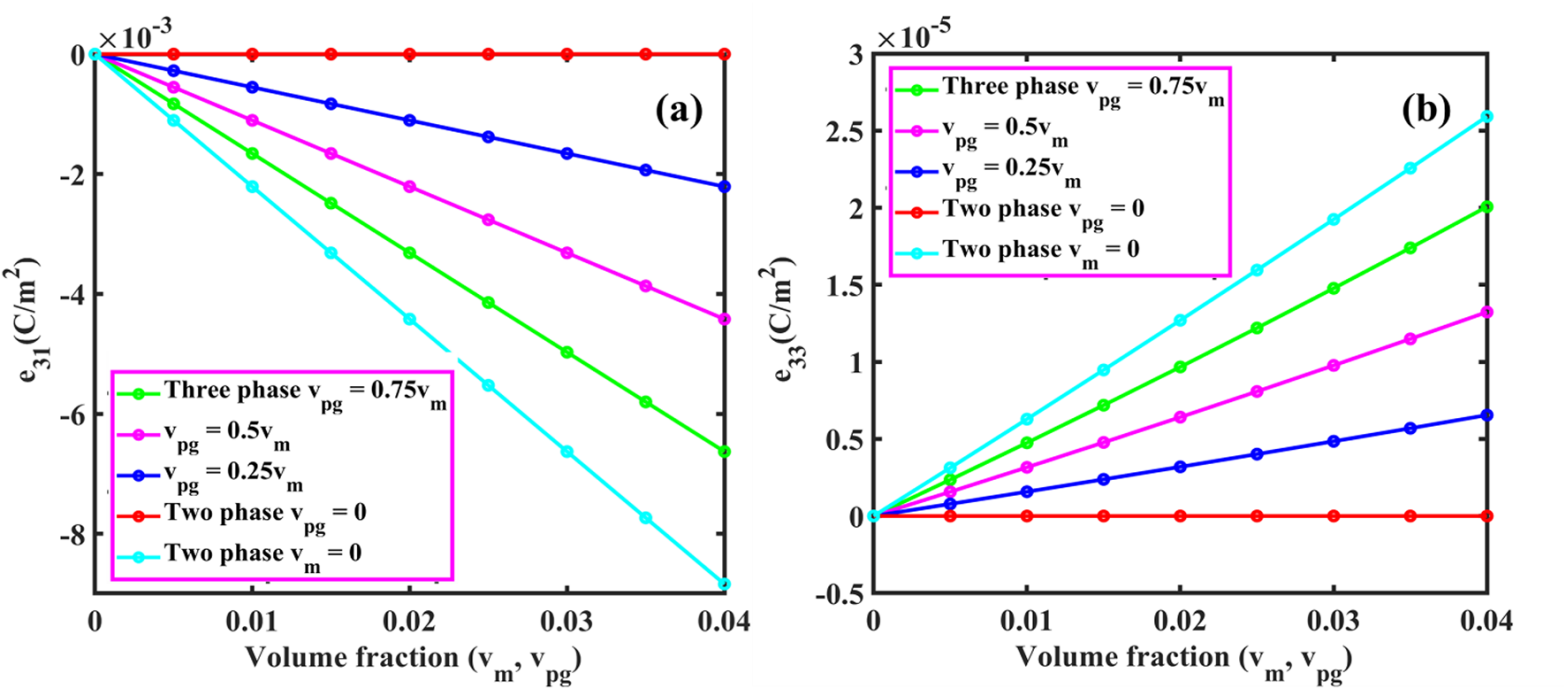
Comparison of effective piezoelectric properties (**a**) $\:{\text{e}}_{31}$ and (**b**) $\:{\text{e}}_{33}$ of MGHPC for different choices of $\:{\text{v}}_{\text{p}\text{g}}$ and $\:{\text{v}}_{\text{m}}$.

Figure 4e depicts the variation of shear coefficients ($\:{\text{C}}_{44}$, $\:{\text{C}}_{55}$, and $\:{\text{C}}_{66}$) concerning $\:{\text{v}}_{\text{p}\text{g}}$ and $\:{\text{v}}_{\text{m}}$ for MRC, GRC, and MGHPC. The proposed micromechanics model neglects boundary conditions, leading to analogous results for all shear constants. Therefore, only the result for the in-plane shear coefficient $\:\left({\text{C}}_{66}\right)$ is presented here. Additionally, these shear properties solely rely on axial and transverse properties. In FE analysis, these shear properties are predicted by applying appropriate support and loading conditions. In Figs. 4 and 5, FE results are not presented for brevity, but both models are in good coherence.

It should be mentioned that GRC and MGHPC are piezoelectric composites due to their active piezoelectric graphene reinforcements. Conversely, MRC without graphene exhibits a passive or non-piezoelectric composite nature. Figure 5(a, b) present the results for effective piezoelectric constants ($\:{\text{e}}_{31}$, $\:{\text{e}}_{33}$) of MRC, GRC, and MGHPC for different choices of $\:{\text{v}}_{\text{p}\text{g}}$ and $\:{\text{v}}_{\text{m}}$. These constants are indicative of the composite’s response to electric fields applied across its thickness, with $\:{\text{e}}_{31}$ and $\:{\text{e}}_{33}$ corresponding to in-plane and out-of-plane actuation, respectively. These piezoelectric properties of the MGHPC were estimated by assuming isofield conditions. It is observed that these piezoelectric properties are increasing with the values of $\:{\text{v}}_{\text{p}\text{g}}$ (Fig. 5a, b). Notably, these effective properties exhibit linear trends for all choices of $\:{\text{v}}_{\text{p}\text{g}}$ and $\:{\text{v}}_{\text{m}}$, and are closely associated with the Voigt upper bound approximation. Although incorporating MXene nanofillers does not influence the piezoelectric constants, adding MXene increases the effective elastic coefficients. These three-phase composites can serve as distributed actuators because of the increased in-plane and out-of-plane actuation constants. In contrast to $\:{\text{e}}_{31}$ and $\:{\text{e}}_{33}$, the value of $\:{\text{e}}_{32}$ of MGHPC become substantially smaller; therefore, the results for $\:{\text{e}}_{32}$ are not presented.

The primary reason behind these improved properties is attributed to the greater surface area of graphene, which facilitates more contact with both nano-reinforcements and matrix, i.e., it enables a strong mechanical interaction between the matrix and nanofillers. With a high surface-to-volume ratio, graphene provides more contact points with the surrounding matrix material, leading to stronger mechanical interaction between the graphene sheets, other nano-reinforcements, and the polymer matrix. This enhanced interfacial bonding between the matrix and the nanofillers contributes to a more effective transfer of stress from the matrix to the reinforcement under mechanical loading. Figures 4 and 5 show that in addition to graphene as reinforcement to the matrix, noteworthy enrichment is noticed due to the addition of MXene. The effective properties of the composite are assessed under two scenarios: one considering the presence of strong covalent bonds, which contribute to strong van der Waals forces between graphene layers/MXene reinforcements and the matrix or contribute to the interaction and in-plane stability of 2D crystalline graphene, and the other without such considerations. Observations demonstrate a substantial augmentation in the overall effective elastic properties of the MGHPC upon the incorporation of 2D graphene and MXene into the epoxy matrix. Therefore, the findings presented in this study pave the way for the development of novel smart structures with enhanced controllability.

Based on these findings, it is recommended that the MGHPC manufacturing process be carefully monitored/controlled to achieve an effective interface. Additionally, optimizing the volume fraction of MXene is essential, as excessive content of nano-reinforcements can lead to brittleness of composites, particularly in scenarios where structural degradation may occur at minimal deformations. From a recent study^12^, we found that the optimal volume fraction for improving the overall mechanical behavior of structures is 0.1% for graphene and 2% for MXene. Hence, the influence of size-dependent properties (i.e., flexoelectricity) on the electromechanical behavior of MGHPC plates is explored. The results were obtained using the improved Kirchhoff’s plate theory and Ritz method and are presented in the following section.

### Coupled electromechanical response of MGHPC structures

**Fig. 6:**
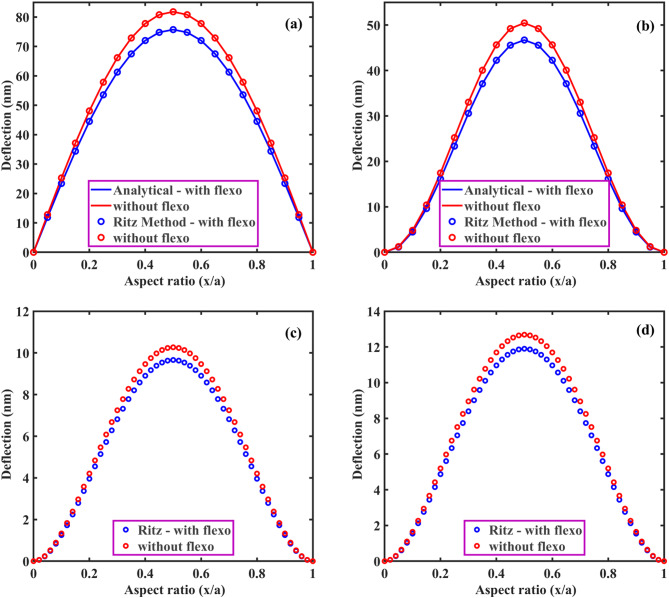
Comparative results for deflection of MGHPC plates under distinct loadings and edge support conditions (**a**) Sinusoidal and (**b**) UDL for SSSS, while only UDL for (**c**) CCCC and (**d**) CCSS $$\:({\text{f}}_{14}=\:{10}^{-9}$$$$\:\text{C}/\text{m})$$.

Now, we discuss the electromechanical static/dynamic response of MGHPC plates with consideration of the effect of flexoelectricity. Figure 6(a-d) present the comparative results for central deflection of MGHPC plates under sinusoidal and uniformly distributed (UDL) loading, considering different edge support conditions like SSSS, CCCC, and CSCS. Specifically, we considered MGHPC plates with dimensions of $$\:h=20\:nm$$ and L = 50 h. Here, we considered the value of the flexoelectric coefficient, i.e., $$\:{\text{f}}_{14}=\:{10}^{-9}$$$$\:\text{C}/\text{m}$$. These results were obtained from the exact Navier’s and the approximate Ritz solutions. All results concerning the plate aspect ratio are presented here. In Fig. 6(a-d), it is evident that regardless of the loading conditions, the maximum deflection is consistently observed at the center of the plates (Eq. 30d). It is seen that the results obtained from the Navier solution and Ritz method match exactly. The Ritz solution converges as the values of N increase. Additionally, it has been observed that even values of N do not enhance the solution beyond what is achieved using the preceding odd value of N. These results demonstrate that the presence of flexoelectricity has a noteworthy influence on the central deflection of plates as compared to deflections without the presence of flexoelectricity. This is mainly because the stiffness of MGHPC plates is increased due to consideration of flexoelectricity. This Navier’s solution cannot be applied for boundary conditions other than SSSS and therefore, the Ritz method is only employed for investigating the deflection of plates considering clamped-clamped (CCCC) and clamped-simply supported (CCSS) edge support conditions, presented in Fig. 6(c-d).

The three-dimensional (3D) plots illustrate the effects of sinusoidal loading with different half-wave numbers (Table 3). The increase in the values of m and n (i.e., 1, 2, 3…) corresponds to a decrease in the bending deflection, as shown in Table 4. Specifically, the center deflection is less for sinusoidal loading than for UDL. This distinction arises from the nature of sinusoidal loading, where the load is distributed as a sinusoidal function (Eq. 30b), resulting in the maximum load acting at the center of the plate while the load is zero at the edges. With increasing m and n (representing the number of half-waves), the deflection reduces because the load distribution becomes more oscillatory, reducing the overall bending in the plate. In contrast, UDL results in a more uniform deflection across the plate compared to a sinusoidal load. This is because a UDL applies a constant load per unit area over the entire plate surface, resulting in a more uniform stress and deflection pattern. Consequently, the impact of sinusoidal loading becomes more pronounced for higher values of m and n.

**Table 4:**
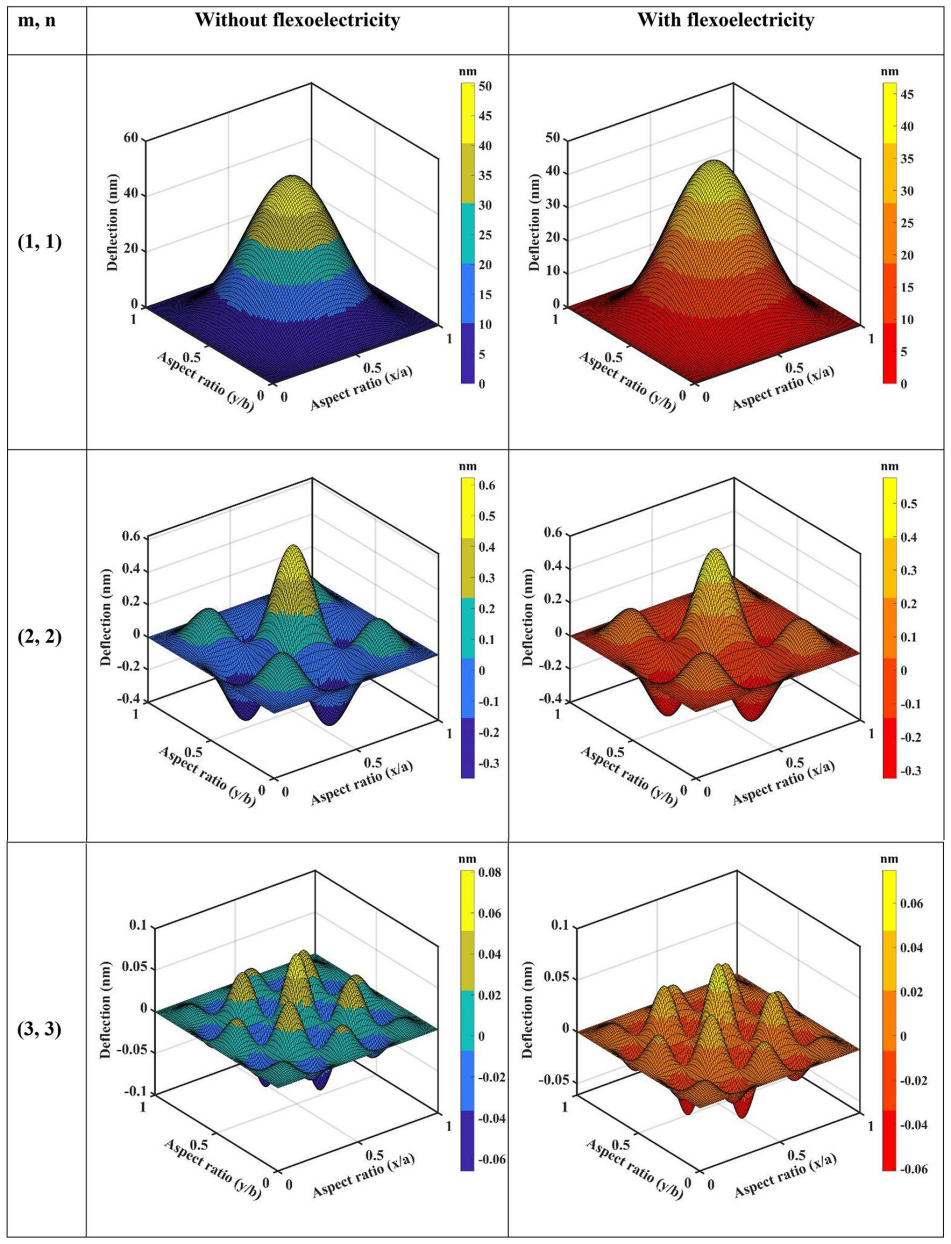
Comparative results for deflection of MGHPC plates under sinusoidal loadings accounting for the effect of half-wave numbers $$\:({\text{f}}_{14}=\:{10}^{-9}$$$$\:\text{C}/\text{m})$$.

**Fig. 7:**
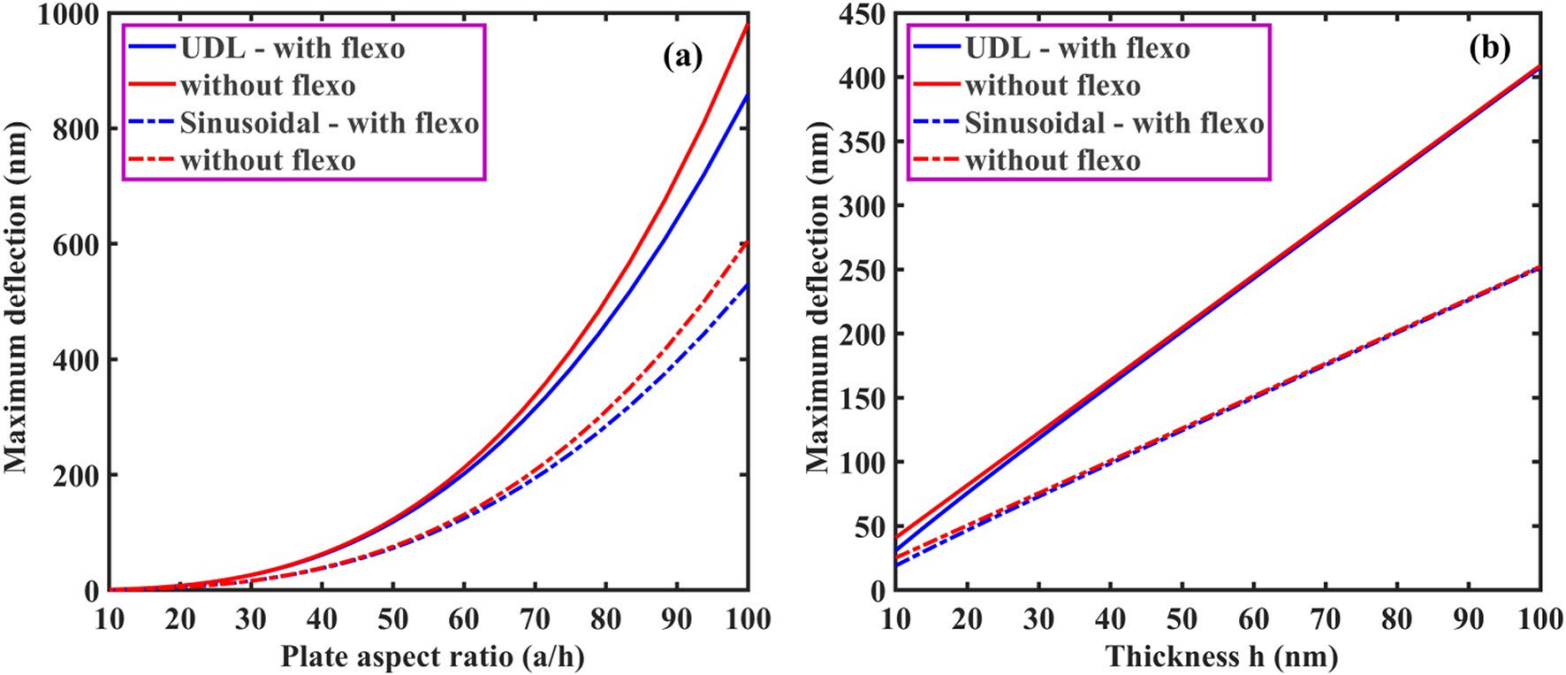
Variation of maximum deflection of MGHPC plate concerning both (**a**) aspect ratio and (**b**) thickness under UDL and sinusoidal loading $$\:({\text{f}}_{14}=\:{10}^{-9}$$$$\:\text{C}/\text{m})$$.

Figure 7 illustrates how the maximum deflection of MGHPC plates varies with changes in plate aspect ratio $\:(a/h)$ and plate thickness $\:\left(h\right)$. In Fig. 7a, the in-plane dimension of the MGHPC plate is constant, $\:a=b=1500$ nm, while in Fig. 7b, the aspect ratio was constrained to $\:a/h=b/h=50$. The flexoelectric effect is particularly pronounced when the plate’s aspect ratio reaches 40 or higher, as shown in Fig. 7a. Notably, flexoelectricity becomes significant when the plates’ in-plane dimensions are within the nanometer scale. When the aspect ratio drops below 30, the plate’s thickness increases relative to its in-plane dimensions, reducing the strain gradients, and thereby minimizing the influence of flexoelectricity. This shows that flexoelectricity is highly size-dependent, with its effect being most significant for thin plates with large in-plane dimensions. Similar results were observed in cases where the diminishing effect of flexoelectricity coincided with increasing plate thickness (Fig. 7b). Moreover, it was apparent that the plate deflection under UDL is greater than sinusoidal loading (Fig. 7a-b). These results emphasize the size-dependent behavior of nanoplates, particularly in terms of flexoelectricity, and how both the plate’s geometry and loading type significantly affect its static response.

**Fig. 8:**
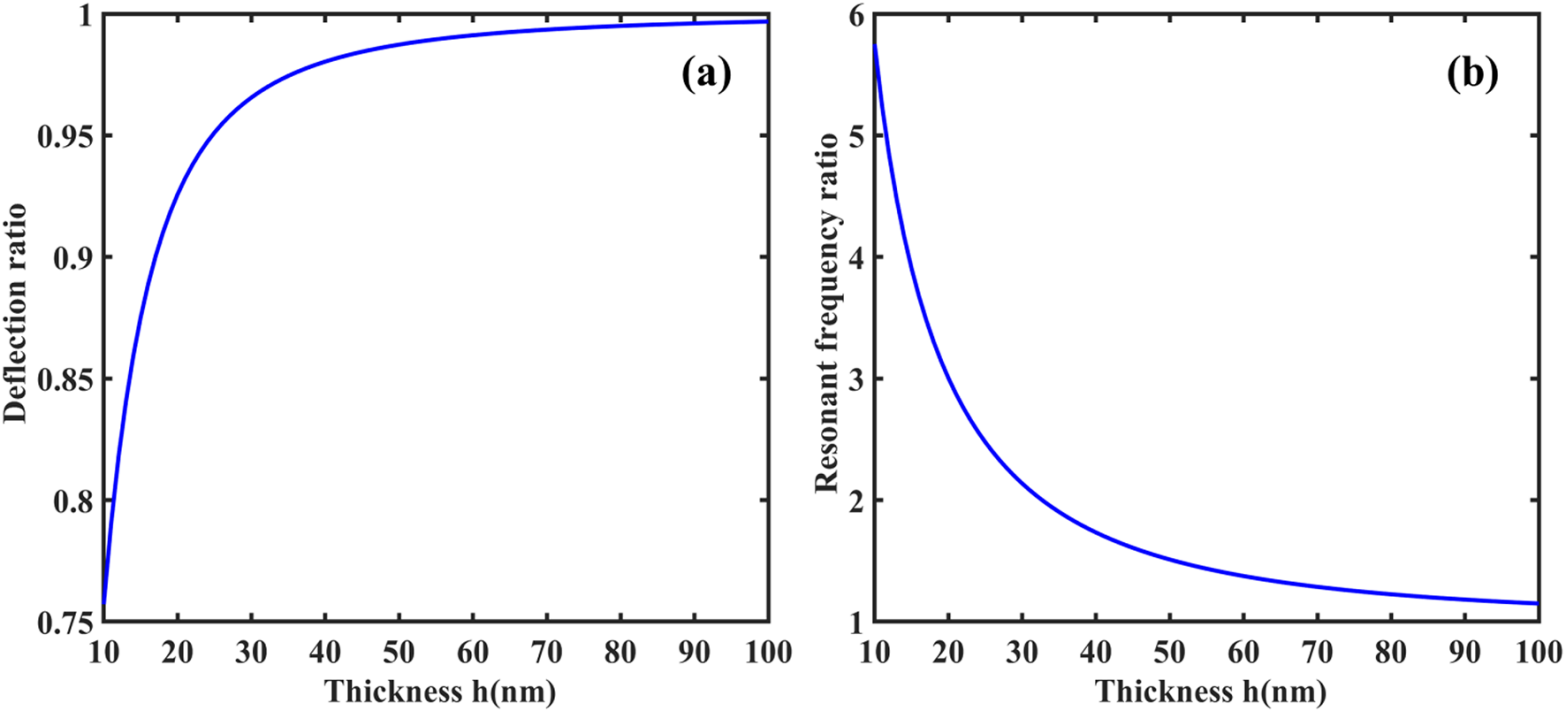
Variation of (**a**) deflection $$\:({\text{f}}_{14}=\:{10}^{-9}$$$$\:\text{C}/\text{m})$$ and (**b**) resonance frequency $$\:({\text{f}}_{14}=\:{10}^{-8}$$$$\:\text{C}/\text{m})$$ ratio of MGHPC plate concerning its thickness.

Figure 8a depicts the fluctuation in deflection ratio in relation to the plate thickness. Here, the deflection ratio is the ratio of the plate deflection, including effects of flexoelectricity $$\:({\text{f}}_{14}=\:{10}^{-9}$$$$\:\text{C}/\text{m})$$, to that obtained without considering the latter effects (i.e., classical results). It can be seen that the influence of flexoelectricity decreases and gradually aligns with classical results as the thickness of the nanoplate increases. Notably, the flexoelectric effect yields results closer to unity at a faster rate when the thickness is larger. Furthermore, the results indicate that flexoelectric effects significantly reduce the deflection of thin nanoplates (i.e., less than 20 nm). This reduction arises from the inherent capacity of flexoelectric effects to enhance the stiffness of the nanoplate (Eq. 26). In other words, the strain gradients, which flexoelectricity relies on, become less pronounced in thicker plates (with greater thickness), reducing the electromechanical coupling that causes additional stiffness. Consequently, the impact of size-dependent phenomena on the electromechanical characteristics of thin nanoplates is substantial. Conversely, analyzing electromechanical characteristics in bulk structures may disregard such effects. We have also investigated the impact of flexoelectricity on the resonance frequency of the MGHPC plates. Figure 8b illustrates the frequency response ratio of the MGHPC plate as a function of the plate thickness $$\:({\text{f}}_{14}=\:{10}^{-8}$$$$\:\text{C}/\text{m})$$. Notably, the size effect becomes apparent for thin plates, with the frequency ratio gradually decreasing and approaching unity. As the plate thickness increases, the strain gradients decrease, reducing the flexoelectric contribution to stiffness, and the resonance frequency approaches the classical value (without flexoelectricity). Hence, size effects become more pronounced at smaller scales and dissipate as the plate size increases.

**Fig. 9:**
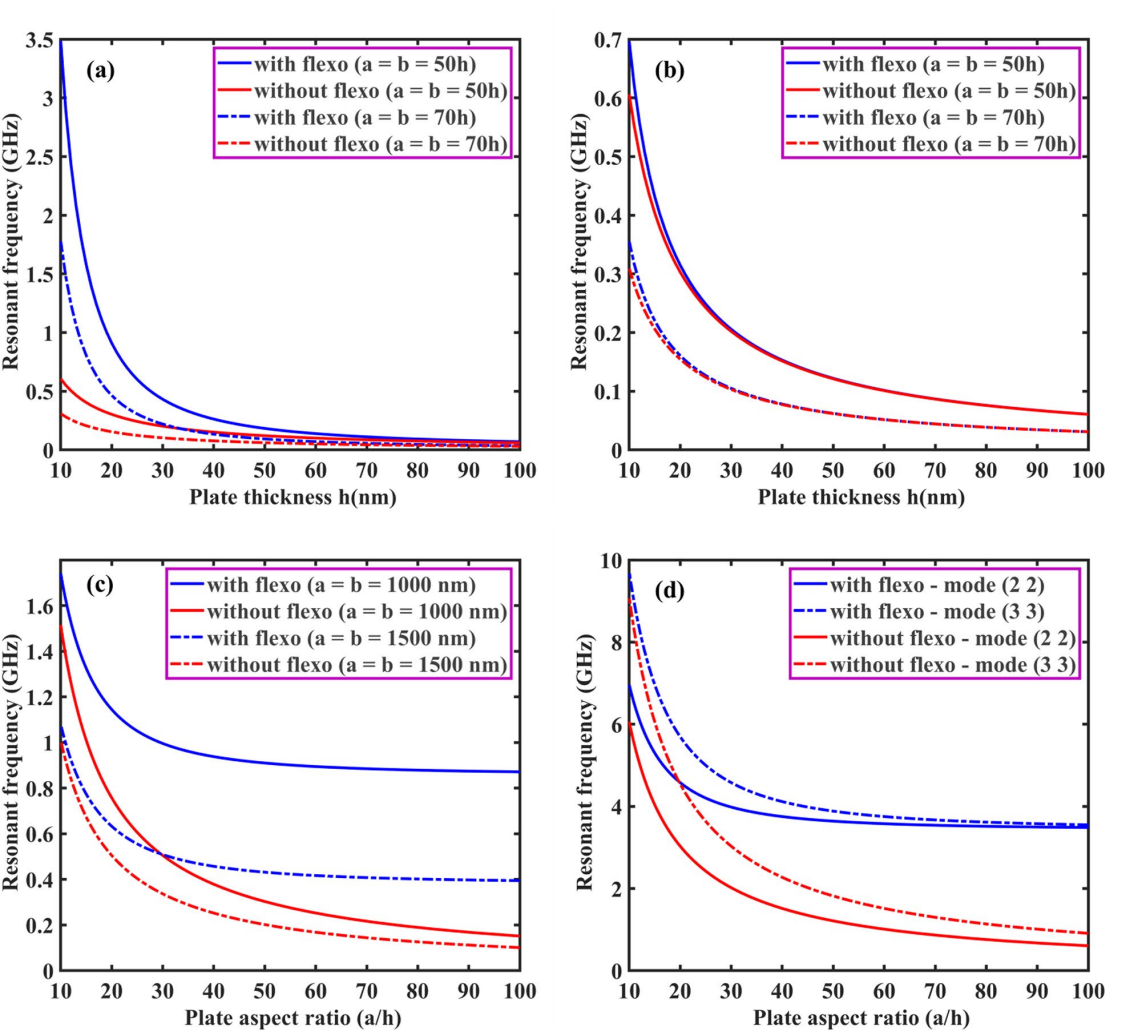
Variation of the resonance frequency considering different plate aspect ratios and flexoelectric coefficients: (**a**) $$\:{\text{f}}_{14}=\:{10}^{-8}\:$$, and (**b**) $$\:{\text{f}}_{14}=\:{10}^{-9}$$ C/m, and different modes: (**c**) mode (1, 1), and (**d**) mode (2, 2); mode (3, 3) $$\:({\text{f}}_{14}=\:{10}^{-8}$$$$\:\text{C}/\text{m})$$.

Figure 9(a-b) show the variation of the resonance frequency of the MGHPC plate with consideration of distinct plate aspect ratios $$\:(\text{a}=\text{b}=50\text{h}\:\text{a}\text{n}\text{d}\:70\text{h})$$ and flexoelectric coefficients (a) $$\:{\text{f}}_{14}=\:{10}^{-8}\:$$and (b) $$\:{\text{f}}_{14}=\:{10}^{-9}$$$$\:\text{C}/\text{m}$$. We varied the value of plate thickness from $$\:10\:\text{n}\text{m}\:\text{t}\text{o}\:100\:\text{n}\text{m}$$. Compared to the static deflection of the plate, the resonance frequency of the MGHPC plate also has a great influence on the flexoelectric effect and plate thickness. The resonance frequency decreases with increasing plate thickness, indicating the size-dependent nature of flexoelectricity. Here, we also considered distinct values of flexoelectric coefficient, i.e., $$\:{\text{f}}_{14}=\:{10}^{-8}\:$$and $$\:{10}^{-9}$$$$\:\text{C}/\text{m}$$. The analysis reveals that the impact of flexoelectricity on the dynamic response of MGHPC plates is small at $$\:{{\text{f}}_{14}=10}^{-9}$$ compared to $$\:{10}^{-8}\:\text{C}/\text{m}$$. It may be due to fact that larger value of flexoelectric coefficient leads to greater stiffness of plates. Consequently, to enhance better result visualization, we opted for a flexoelectric coefficient value of $$\:{10}^{-8}$$$$\:\text{C}/\text{m}$$ and examined, in more detail. Figure 9(c-d) represents the variation of the resonance frequency of the MGHPC plate concerning the plate aspect ratio considering different in-plane dimensions and modes such as mode (1, 1), (2, 2), and (3, 3). We considered the in-plane dimensions of the plate as $$\:1000\:\text{n}\text{m}\:\text{a}\text{n}\text{d}\:1500\:\text{n}\text{m}$$ and varied plate thickness for mode (1, 1), as shown in Fig. 9c. The flexoelectricity effect reduces with increasing in-plane dimensions, indicating the resonance frequency dependence on in-plane dimensions (Refer Eqs. 33b and 55). In other words, the resonance frequency is directly proportional to mode numbers (i.e., m and n) while inversely proportional to in-plane dimensions. Figure 9d shows that the resonance frequency increases for greater modes like modes (2, 2) and (3, 3). In summary, the flexoelectric effect is particularly pronounced for smaller plate thicknesses at the nanoscale and cannot be disregarded.

The practical implementation of the nano-scaled structures is critically important, as highlighted in this paper. In the view of experimental studies, finding an excellent fabrication technique for multiscale or nanocomposite structures is crucial. Nanofabrication methods like layer-by-layer (LbL) assembly, dispersion, and solution blending are widely used to fabricate multifunctional thin films^15^. Cost-effective techniques like spray coating, solvent casting^16^, and hydrothermal synthesis followed by freeze-drying^17^ were specifically used for graphene-MXene-based composites. For instance, assemblies of graphene oxide (GO) and polyethylenimine (PEI) can be tailored to a thickness of ~ 4.5-5 nm by adjusting GO layers. Other studies have achieved 8–10 nm thickness using 4 to 30 graphene platelets^66–69^. These techniques allow nanocomposite fabrication at the nanometer scale. Therefore, one can use these techniques to fabricate graphene-MXene-based nanocomposite and achieve significant electromechanical response considering flexoelectric effects. In the view of analytical and numerical study, in the present study, we considered ‘classical Kirchhoff plate theory’ (Eq. 7), which is accurate for thin plates ($\:a/h\:\ge\:20$). For example, a square plate in this study with an aspect ratio of $\:(a/h\:=50)$ and thickness of 20 nm can easily incorporate graphene sheets of nanometer thickness.

## Potential perspective towards engineering applications

These proposed nanocomposite structures combine piezoelectric materials with nano-reinforcements to produce a class of materials with exclusive potential applications. This work presents an analysis using an effective analytical and approximate model. In the following, we discuss important real-world engineering applications related to piezocomposites.

### Smart materials and structures

The hybrid piezocomposite material described here has potential applications in advancing smart structures. These materials may detect alterations in the operational environment by leveraging their piezoelectric properties. This versatility allows diverse applications, including actuators, shape adaptation, active vibration control, and mechanical properties. In micro- and nano-electromechanical systems (M/NEMS), flexoelectric piezocomposites can be employed to fabricate miniature sensors, actuators, and resonators, catering to a range of sectors such as mechanical, electronics, aircraft, and medical industries.

### Vibration damping and control

The hybrid piezocomposite material can be effectively utilized in the automotive, civil, and aerospace sectors to control and dampen vibration. For example: For minimizing vibrations and mitigating resonance in structures such as car panels, aircraft wings, and buildings, these tailored materials with specific dampening properties prove valuable.

### Structures with shape morphing abilities

When subjected to electrical stimuli, a hybrid piezocomposite material can be designed to undergo shape transformations. These materials can be applied in aerospace engineering, contributing to the creation of adaptive structures and morphing surfaces that can enhance aerodynamic performance. Comparable applications are observed in diverse mechanical systems.

### Energy storage and absorption

Using hybrid piezocomposite materials can be implemented in different applications like automotive crash pads and helmet liners for absorbing/minimizing impact. Integrating nano-reinforcements facilitates effective energy dissipation during impact events, minimizing potential injury and damage through flexoelectric energy conversion.

### Structural health monitoring (SHM)

The hybrid piezocomposite material discussed here can be effectively applied for SHM. These materials can detect system property alterations by incorporating sensors that harness the piezoelectric effect, enabling real-time structural damage or defect detection.

### Soft robotics integration

The hybrid piezocomposite material can be seamlessly combined with soft robotic systems, resulting in deformable structures. Such materials contribute to developing adaptable and flexible robots, particularly suitable for delicate activities that improve the human-robot interface.

## Conclusions

The present study presents analytical models designed to analyze the electromechanical response of MXene/graphene-based hybrid piezocomposite (MGHPC) plates, incorporating the flexoelectric effect. Specifically, the static/dynamic behavior of MGHPC plates, including deflection and natural frequency, were investigated for various edge support conditions (clamped-clamped, simply supported, and clamped-simply supported) and loadings (sinusoidal and uniform distributed loadings). Additionally, the modified Ritz method was used to obtain approximate solutions found to be in agreement with the exact analytical solutions. The current research study yields several significant findings, as outlined in the following.

- The effective piezoelastic properties determined using two- and three-phase micromechanical models for MGHPCs agree with FE predictions and earlier reported experimental results.
- The micromechanical study shows that adding 2D MXene and graphene as nano-reinforcements in an epoxy matrix substantially augments the effective properties of MGHPCs.
- The static deflection of MHGPC plates decreases significantly with the incorporation of flexoelectricity, regardless of the loading and support conditions when compared to piezoelectric plates.
- Further, the study investigates the electromechanical response of nanoplates. It considers various critical parameters, including aspect ratios, plate thickness, flexoelectric coefficients, and graphene/MXene volume fractions.
- The impact of flexoelectricity on electromechanical characteristics, including deflection and natural frequency, is more significant in nanoplates with smaller thicknesses. As the plate thickness increases, this response gradually diminishes. These findings reveal a new size-dependent behavior that holds significance for the development of novel M/NEMS.

This study shows that when combined with MXenes, graphene nano-reinforcements can significantly increase the strength/stiffness of multifunctional composites, broadening the range of applications for these materials. In the context of future applications, by harnessing the impressive mechanical properties and electrical conductivity of MXene, these types of novel hybrid polymer piezocomposites can find utility in flexible electronics and high-strength structural applications (as electric properties of MXene are not considered herein). These composites may be implemented for real-time monitoring of structural integrity, providing an added layer of functionality. Computational analysis results highlight the significant potential of 2D graphene and MXene nano-reinforcements in creating innovative multifunctional composites with outstanding mechanical properties.

## Data Availability

The datasets used and/or analysed during the current study available from the corresponding author on reasonable request.
